# Benzenetriol-Derived Compounds against Citrus Canker

**DOI:** 10.3390/molecules26051436

**Published:** 2021-03-06

**Authors:** Lúcia Bonci Cavalca, Ciaran W. Lahive, Fleur Gijsbers, Fernando Rogério Pavan, Dirk-Jan Scheffers, Peter J. Deuss

**Affiliations:** 1 Department of Molecular Microbiology, Groningen Biomolecular Sciences and Biotechnology Institute, University of Groningen, 9747 AG Groningen, The Netherlands; l.bonci.cavalca@rug.nl (L.B.C.); fleurgijsbers@gmail.com (F.G.); 2 Department of Chemical Engineering (ENTEG), University of Groningen, Nijenborgh 4, 9747 AG Groningen, The Netherlands; c.w.lahive@rug.nl; 3 School of Pharmaceutical Sciences, São Paulo State University (UNESP), Araraquara 14800-903, Brazil; fernando.pavan@unesp.br

**Keywords:** *Xanthomonas citri*, antimicrobials, lignocellulosic biomass, bio-based chemicals, phenolic compounds

## Abstract

In order to replace the huge amounts of copper salts used in citrus orchards, alternatives have been sought in the form of organic compounds of natural origin with activity against the causative agent of citrus canker, the phytopathogen *Xanthomonas citri* subsp. *Citri*. We synthesized a series of 4-alkoxy-1,2-benzene diols (alkyl-BDOs) using 1,2,4-benzenetriol (BTO) as a starting material through a three-step synthesis route and evaluated their suitability as antibacterial compounds. Our results show that alkyl ethers derived from 1,2,4-benzenetriol have bactericidal activity against *X. citri*, disrupting the bacterial cell membrane within 15 min. Alkyl-BDOs were also shown to remain active against the bacteria while in solution, and presented low toxicity to (human) MRC-5 cells. Therefore, we have demonstrated that 1,2,4-benzenetriol—a molecule that can be obtained from agricultural residues—is an adequate precursor for the synthesis of new compounds with activity against *X. citri*.

## 1. Introduction

Citrus canker is a bacterial plant disease caused by the pathogen *Xanthomonas citri* subsp. *citri* that affects all cultivated citrus species, for example, oranges or lemons. This disease causes lesions in the aerial tissues of citrus plants, generating spots and, in more severe cases, the loss of leaves and premature fruit drop, being responsible for economic losses in agriculture and restrictions to international trade [1]. Citrus canker is believed to have originated in Asia and is nowadays also present in Africa, Oceania, and in the Americas, where it is already considered endemic to some of the main citrus-producing areas, such as São Paulo state (Brazil) and Florida (USA).

**Figure 1:**
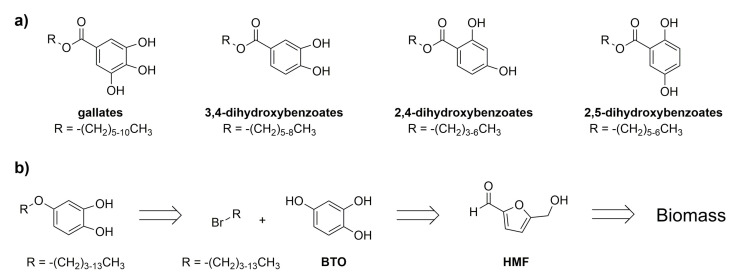
(**a**) Reported organic compounds with activity against *Xanthomonas citri* [3,4,5]; (**b**) Retrosynthetic analysis (design strategy) for novel BTO (bio)-based agents against citrus canker. BTO: 1,2,4-benzenetriol; HMF: hydroxymethylfurfural.

Huge amounts of copper salts are sprayed annually in citrus orchards as the main strategy for controlling the disease. This process threatens the environment and human health due to its toxic and cumulative effects [2]. As an alternative to copper salts, organic compounds from natural sources have been investigated, and have so far shown promising activity against the plant pathogen [3,4,5]. In recent years, it has been demonstrated that several alkylated compounds derived from hydroxybenzoic acids such as gallic, β-resorcylic, gentisic, and protocatechuic acids are capable of completely inhibiting *X. citri* (Figure 1a). All of these new compounds with anti-*X. citri* activity have similar structures, being formed by esterification between benzoic acid derivatives with two or three hydroxyls (phenols) and a linear aliphatic chain of up to 11 carbons.

Taking into account that the antibacterial activity of these compounds seems to be directly related to the presence of the phenols and the linear aliphatic carbon chain, we designed a new series of compounds to be synthesized using 1,2,4-benzenetriol (BTO, Figure 1b). BTO was demonstrated to be attainable from biomass by hydrothermal reaction of hydroxymethylfurfural (HMF) in the presence of catalysts [6,7]. HMF has been reported as a key bio-based platform chemical and is projected to be available at low price once produced at large scale from agricultural residues [8,9]. BTO is composed of an aromatic ring with three phenolic hydroxyls, of which the one in the 4 position can be selectively modified to obtain ethers (Figure 1b) [10]. Lateral carbon chains from 4 to 14 carbons were chosen. These compounds were tested for *X. citri* in order for us to provide a range which could allow us to determine optimal lateral chain length and possible correlations between antibacterial activity and carbon chain size. Moreover, in the designed molecules the aromatic group was linked to the alkyl chain by an ether bond instead of an ester bond, allowing comparisons between both bonds and the identification of possible roles for these bonds in the antibacterial activity of these compounds. Furthermore, the potential activity against other bacteria was investigated as well as their mode of action and toxicity to mammalian cells.

## 2. Results and Discussion

### 2.1. A Three-Step Synthesis Route from BTO to 4-Alkoxy-1,2-Benzenediols (Alkyl-BDOs)

To obtain the new compounds based on 1,2,4-benzenetriol, we were first interested in a one-pot synthesis catalyzed by 1,8-diazabicyclo[5.4.0]undec-7-ene (DBU) as this could be considered to provide an easy route to the proposed set of compounds [10]. However, this route proved to be inconvenient as, in contrast to the earlier report [10], the etherification of BTO showed no clear preference for substituting the position 4 selectively, resulting in a mixture of products etherified in any one, two, or all three positions. Only very low yields (<1%) of individual compounds were achieved after separation of the product mixture by chromatography. Furthermore, the correct identification of each product as to the position(s) to which the carbon chain was added proved to be a challenge, leading to us not pursuing this route further.

**Scheme 1:**
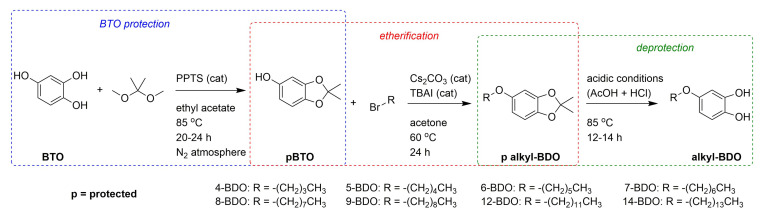
Three-step synthesis of 4-alkoxy-1,2-benzenediols (alkyl-BDOs) from 1,2,4-benzenetriol (BTO). pBTO: protected BTO (2,2-dimethylbenzo[d][1,3]dioxol-5-ol); p alkyl-BDO: protected alkyl-BDO (2,2-dimethyl-5-(alkyloxy)benzo[d][1,3]dioxole).

As an alternative, a three-step synthetic route consisting of BTO protection [11], etherification, and deprotection was designed (Scheme 1) consisting of: (a) protection of BTO; (b) purified protected BTO (pBTO) etherification; and (c) crude protected alkylated BTO deprotection and purification. This route achieved target compound synthesis with a reasonable overall yield of up to 9% for the alkyl-BDOs (Table S1, in SI); the main challenge of this approach was to obtain protected BTO, which was achieved with a final yield of 30% (after purification). Our attempts showed that the presence of oxygen or a high substrate concentration in the reaction favored the formation of byproducts that were related to the tendency of BTO to dimerize as we previously reported [7,12]. Moreover, BTO protection only occurred in the presence of a large excess of protecting agent (2,2-dimethoxypropane), and ethyl acetate proved to be a more suitable solvent than toluene for this reaction. The steps of etherification and deprotection were straightforward and etherification was also shown to be suitable for other phenolic substrates, such as sesamol. Although not ideal in terms of green chemistry, this route allowed us to obtain a sufficient quantity of these compounds from BTO for an initial evaluation, keeping open the possibility of returning to optimization of this synthetic route in case the compounds are deemed of interest for further studies. Synthesis routes using other starting materials have been previously described to obtain some of the target compounds reported here. The synthesis of 4-alkoxy catechols and other catechol derivatives from 4-butoxyphenol and 3,4-dihydroxybenzaldehyde was described respectively by [13,14,15].

### 2.2. Alkyl-BDOs Inhibit Bacterial Cell Growth

The ability of alkyl-BDOs with varying alkyl chain lengths to inhibit bacterial cell growth was initially assessed by monitoring the growth of *Xanthomonas citri* subsp. *citri* and *Bacillus subtilis* liquid cultures treated with the compounds at different concentrations. *B. subtilis* is used as a model to test for activity against Gram-positive bacteria. Alkyl-BDOs with lateral chains ranging from five to eight carbons completely inhibited both *Xanthomonas citri* subsp. *citri* and *Bacillus subtilis* cell growth for 24 h at the concentration of 100 µg·mL^−1^ (see Supporting Information S2). The inhibitory effect was strong at the concentration of 50 µg·mL^−1^ and there was a mild effect at 12.5 µg·mL^−1^, showing a clear dose–response correlation. The presence of alkyl-BDOs caused a reduction in the maximum optical density (OD) of bacterial populations and a delay in the initiation of exponential growth phase. 

Although the method does not allow a precise quantification of this inhibitory effect, we can observe that (a) at 100 µg·mL^−1^, although 9-BDO initially shows an inhibitory effect, *X. citri* is able to start bacterial growth after ~12 h of treatment; (b) at 50 µg·mL^−1^, the growth curve of *X. citri* treated with 9-BDO already shows a slope similar to the control curve (delayed by about 9 h), where *X. citri* treated with 8-BDO starts growth after ~12 h and only the compounds with 4 to 7 carbons in their lateral chain are capable of maintaining total inhibition over 24 h; (c) at 25 µg·mL^−1^, the growth curves of *X. citri* treated with 4- and 5-BDO also resemble the control curve (delayed by about 6 h) and *X. citri* treated with 6-, 7- and 8-BDO start growth after ~9 h, without reaching exponential phase; and (d) at 12.5 µg·mL^−1^, all growth curves eventually reach maximum OD, with treatment with 7-BDO notably causing the longest delay in the onset of bacterial growth. It is notable that, while 7-BDO is one of the most active compounds, its protected intermediate p7-BDO does not exert any inhibitory activity against the tested bacteria, confirming the essential role of the two phenolic groups in the activity of these molecules, as also observed for dihydroxybenzoates and dihydroxyphenyl alkanoate [4,16]. 

### 2.3. Alkyl-BDOs Have Bactericidal Activity

Alkyl-BDO activity against Gram-negative (*Xanthomonas citri* subsp. *citri* and *Escherichia coli*) and Gram-positive bacteria (*Bacillus subtilis* and *Lactococcus lactis*) was further assessed and the minimum inhibitory concentrations (MICs) determined by broth microdilution method with concentrations ranging from 100 to 12.5 µg·mL^−1^. *X. citri*, *B. subtilis*, and *L. lactis* showed susceptibility (MIC up to 100 µg·mL^−1^) to all alkyl-BDOs with lateral chains ranging from 5 to 8 carbons (Table 1). Therefore, we found no evidence of specificity for Gram-positive or -negative bacteria by these compounds. The minimum inhibitory concentration of alkyl-BDOs against *X. citri* is within the same range as the MIC found for commercially available copper oxychloride (43.1 µg·mL^−1^) [4].

The minimum bacteriostatic/bactericidal concentration (MBC) assay results confirmed the bactericidal nature of alkyl-BDO´s inhibitory activity (see Supporting Information S2), showing complete absence of bacterial growth after treatment with the active compounds. As expected, the positive control (kanamycin, 20 µg·mL^−1^) showed a total absence of bacterial growth, while there was no growth inhibition in the negative control with dimethyl sulfoxide (DMSO 1% *v*/*v*). Therefore, the bactericidal activity of alkyl-BDOs was shown to be similar to that of di and trihydroxybenzoates, with only 8- and 9-BDOs being slightly less active than the corresponding esters (Figure 1a). Despite this small difference, there is no evidence that the ester or ether groups could play a significant role in the activity of these molecules, since the MIC/MBC values determined for both series were very similar, especially when compared to the variations observed between compounds with carbon chains of different sizes.

As observed for dihydroxybenzoates [4], alkyl-BDOs with side chains longer than 7 carbons exhibited little or no activity, as phenolic compounds with strongly hydrophobic tails tend to have their solubility reduced in the culture medium, preventing them from crossing the bacterial cell membrane. In addition, although compounds 4- to 7-BDO have an identical MIC value, those with less than seven carbons in their side chain did not have as strong and prolonged an effect as 7-BDO at lower concentrations (see item 2.2), indicating that the ideal length of the side chain for optimal alkyl-BDOs activity against *X. citri* is seven carbons.

### 2.4. Alkyl-BDOs Permeabilize the Bacterial Cell Membrane

**Figure 2:**
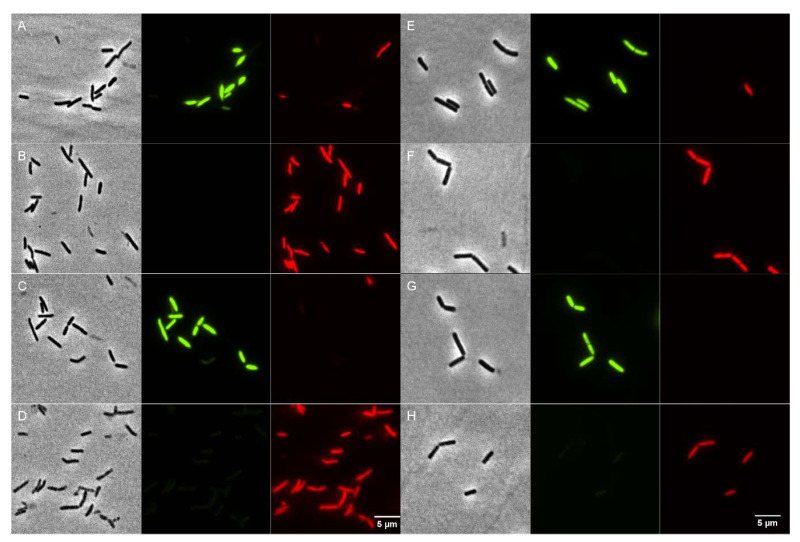
Fluorescence microscopy images of *X. citri* (**A**–**D**) and *B. subtilis* (**E**–**H**) treated with p7-BDO at 100 µg·mL^−1^ (**A**,**E**), 7-BDO at 50 µg·mL^−1^ (**B**,**F**), negative control (**C**,**G**), and positive control (**D**,**H**). Panels show, respectively, phase contrast, FITC, and TRITC images.

To investigate the mode of action of the active alkyl-BDOs, the membrane integrity of *X. citri* and *B. subtilis* cells was evaluated by staining with nucleic acid dyes (Syto9 and propidium iodide) and fluorescence microscopy. Syto9 crosses intact membranes and stains all cells, while propidium iodide can only penetrate cells with a permeabilized membrane and quenches Syto9, allowing the identification of both intact and permeabilized cells. Imaging of *X. citri* and *B. subtilis* treated with the intermediate compound p7-BDO (Figure 2A,E) and compound 7-BDO (Figure 2B,F) showed that while the intermediate does not disturb the membrane in the majority of cells, compound 7-BDO was able to permeabilize cells of both Gram-negative and Gram-positive bacteria within 15 min of treatment (Table 2). Panels C and G in Figure 2 correspond to the negative control cells with intact membrane and panels D and H to positive control cells with permeabilized membrane. Membrane disruption has also been highlighted as the main mode of action of phenolic molecules with antimicrobial activity by several other studies [17,18,19,20,21], including anti-*X. citri* compounds previously reported by our group [3,4,22,23].

### 2.5. Alkyl-BDOs Remain Active against X. citri While in Solution

Taking into account that active compounds against citrus canker are sprayed in aqueous solution [24] combined with our earlier observation of decomposition pathways for BTO [7], the ability of alkyl-BDOs to keep their antibacterial activity while stored in a solution was assessed. This was done by comparing the MICs initially determined to MICs observed in broth microdilution assays performed with compounds pre-incubated for 24 h at 30 °C prior to inoculation. After pre-incubation, all alkyl-BDOs kept the same level of activity against *X. citri* but were no longer able to inhibit *B. subtilis* growth (Table 3). In addition to possible discrepancies caused by the metabolism of each species, the standard growth media used to cultivate and determine the MIC of *X. citri* and *B. subtilis* also differ in composition. As a standard, *X. citri* is grown in nitrogen–yeast–glycerol medium (NYG) medium and *B. subtilis* in Lysogeny broth (LB)–Lennox, which could be the cause for the total loss of activity of alkyl-BDOs against *B. subtilis* in contrast to the maintained effect against *X. citri*.

Since the results observed in the broth microdilution assays performed with compounds’ pre-incubation suggested that medium composition could be a factor affecting the ability of alkyl-BDOs to retain their antibacterial activity in solution, the test was then applied for the same bacteria (*X. citri*) in different media, allowing for a comparison of possible medium composition effects. As observed with *B. subtilis*, all alkyl-BDOs had their activity reduced against *X. citri* when pre-incubated for 24 h in LB–Lennox medium (Table 4), while the same does not happen in NYG medium, suggesting that the differences in composition between LB–Lennox and NYG media should have an influence over the stability of the compounds. The main differences between both media are that NYG is composed of 2% glycerol and LB–Lennox has a NaCl concentration more than 60 times higher than that of the NYG medium; the relatively high salt concentration in LB–Lennox may promote the aggregation of the compounds and reduce their availability in the medium over time.

### 2.6. Alkyl-BDOs Cytotoxicity

The cytotoxicity of compounds 6-BDO, 7-BDO, and 8-BDO against two mammalian cell lines was determined by the resazurin reduction method. While none of the tested compounds were toxic against the rat ascite macrophage cell line J774A.1, they all showed low toxicity to human lung fibroblast cells MRC-5 (Table 5) when compared to doxorubicin, a widely used chemotherapy medication [25]. The toxicity levels shown by alkyl-BDOs against MRC-5 cells are similar to those reported for other polyphenolic molecules with promising activity against *Xanthomonas citri* subsp. *citri*, namely alkyl gallates [26] and dihydroxybenzoates [4]. Some gallates (propyl, octyl, and dodecyl) and other similar compounds (alkyl hydroxybenzoates) are regulated by the U.S. Food and Drug Administration (FDA) as food additives for human consumption, being used by the food industry as antioxidants, flavorings/adjuvants or antimicrobials; therefore, alkyl-BDOs should also be expected to be safe for application in the field or even for contact with food.

## 3. Materials and Methods

### 3.1. Chemicals and Analytical Equipment

All reagents and solvents were acquired from commercial courses using the highest available purity; BTO (95%, Fluorochem), ethyl acetate (≥99.8%, ChromAR^®^), pyridinium p-toluenesulfonate (≥99%, Sigma-Aldrich), silica for column chromatography (FlashPure 12 g, Büchi), acetone (≥99.5%, ChromAR^®^), by cesium carbonate (99%, Sigma-Aldrich), tetrabutylammonium iodide (≥99%, Sigma-Aldrich) High Resolution Mass Spectrometry (HRMS) and Fourier Transform Infrared Spectroscopy (FTIR). NMR and FTIR spectra and compounds’ characterization can be found in the SI. ^1^H and ^13^C NMR spectra were recorded on an Agilent Technologies 400 MHz NMR Premium Shielded Magnet using DMSO-d6 as solvent at room temperature. HRMS were measured with an Thermo LTQ Orbitrap XL with ESI ionization; samples were injected and eluted with 0.15 mL/min acetonitrile + 0.1% NH_3_. FTIR analyses were performed on Shimadzu IRTracer-100 equipment with ATR Specac Golden Gate KRS5. Melting points were determined with Büchi Melting Point M-560 equipment.

### 3.2. Synthesis, Purification, and Characterization of Alkyl-BDOs

The general protocol for the synthesis of a series of new phenolic compounds based on 1,2,4-benzenetriol was established as a three-step route: (a) protection of BTO was achieved by refluxing 7.9 mmol of BTO with 117.2 mmol of dimethoxypropane in 250 mL of ethyl acetate, catalyzed by pyridinium p-toluenesulfonate (0.44 mmol) for 20 to 24 h ([11], with modifications) under a nitrogen atmosphere, followed by extraction with ethyl acetate/water and purification by silica column (heptane/3–5% gradient of EtOAc); (b) protected BTO was etherified under normal atmosphere by refluxing 2.4 mmol with 3.6 mmol of alkyl bromide in 36 mL of acetone and catalyzed by cesium carbonate (0.36 mmol) and tetrabutylammonium iodide (3.1 mmol) for 24 h, followed by the solvent evaporation and extraction with diethyl ether/water; and (c) deprotection of crude BTO derived ethers was achieved under normal atmosphere at acidic conditions by refluxing the product for 12 to 14 h in 7.5 mL of acetic acid added of 2.5 mL of HCl 3 M, followed by solvent evaporation, extraction with diethyl ether/water and purification by silica chromatography (heptane/7–12% gradient of EtOAc). The progress of each reaction was verified by thin layer chromatography (7:3 n-heptane:ethyl acetate) and the products were characterized by ^1^H and ^13^C Nuclear Magnetic Resonance (NMR)., 

### 3.3. Bacterial Strains and Standard Growth Conditions

The Gram-negative and Gram-positive bacteria tested and the standard growth conditions used are summarized in Table 6. *Xanthomonas citri* subsp. *citri* 306 is the pathogenic strain sequenced and described by the São Paulo State consortium [27] and was kindly provided by Dr. H. Ferreira (UNESP, Rio Claro/SP, Brazil). The other strains are all from our laboratory collection and are available on request.

### 3.4. Bacterial Growth Inhibition Assessment

*X. citri*, *B. subtilis, E. coli*, and *L. lactis* were cultivated overnight, diluted in fresh medium (Table 6) to an optical density at 600 nm (OD600) of ~0.1, incubated until OD600 = 0.3–0.4, and then diluted again 100 times in fresh medium. Aliquots with 100 µL of culture dilutions treated with alkyl-BDOs at concentrations ranging from 100 to 12.5 µg·mL^−1^ were distributed in a 96-well microplate and incubated at 30 °C during 24 h with constant agitation. The minimum inhibitory concentrations (MICs) were determined by broth microdilution method as the lowest concentration in which bacterial growth could not be detected after 24 h incubation. To assess the nature of alkyl-BDOs inhibitory activity, the minimum bacteriostatic/bactericidal concentration (MBC) method was applied by transferring a few microliters of each well (after incubation with the compounds) to an agar plate without compound followed by incubation under standard conditions to allow bacterial growth. MIC and MBC values were established according to the results of three biological replicates. The broth microdilution method provides a range for the MIC and MBC (for example, between 25 to 50 µg·mL^−1^) and, as standard, the higher value of this range is defined as the MIC/MBC. To follow growth inhibition in real time, *X. citri* and *B. subtilis* were precultured as described, the 96-well microplate was incubated at 30 °C during 24 h with constant agitation in a Synergy Power Wave reader (BioTek), and OD600 was measured every 30 min. For every condition, three cultures were monitored. Plots of the growth curves (mean of three with SD indicated) were prepared using the software Graphpad Prism 6. Stock solutions were prepared in DMSO and the growth conditions were performed as indicated in Table 6. Kanamycin (20 µg·mL^−1^), and DMSO 1% (*v*/*v*) were used as positive and negative controls, respectively.

### 3.5. Membrane Integrity Evaluation

Cell membrane integrity was assessed by fluorescence microscopy. Fluorescent dyes SYTO9 and propidium iodide were applied prior to visualization following instructions for Live/Dead BacLight bacterial viability kit (Molecular Probes L7007). *X. citri* and *B. subtilis* cells were harvested at OD600 = 0.3–0.4 and treated with alkyl-BDOs for 15 min at 30 °C. DMSO 1% was used as negative control, heat shock (65 °C for 15 min) was used as positive control for *X. citri* and nisin (5 µg·mL^−1^) as positive control for *B. subtilis*. Cells were immobilized in agarose layer and observed with Nikon Ti microscope equipped with an ORCA-flash 4.0 camera (Hamamatsu) and filters FITC and TRITC. Images were acquired with NIS Elements 4.10 software and processed using ImageJ 1.52p.

### 3.6. Alkyl-BDOs Stability

Broth microdilution plates with alkyl-BDOs dilutions ranging from 100 to 12.5 µg·mL^−1^ were prepared and pre-incubated for 24 h at 30 °C with constant agitation prior to *X. citri* and *B. subtilis* inoculation. Broth microdilution assay was then performed as described in Section 3.3, following the standard growth conditions for each bacteria. The results were compared to MICs determined in the same medium without pre-incubation. To evaluate the influence of medium composition on alkyl-BDOs stability, the same test was applied to *X. citri* in both NYG and LB–Lennox media. 

### 3.7. Determination of the Cytotoxicity Index (IC50) 

The cytotoxicity index (IC50) for three alkyl-BDOs (6-BDO, 7-BDO, and 8-BDO) was determined by metabolic reduction of resazurin. Mammalian cell lines MRC-5 (ATCC CCL-171) and J774A.1 (ATCC TIB-67) were cultivated respectively in DMEM (GIBCO^®^) and RPMI (GIBCO^®^) media supplemented with 10% fetal bovine serum and 1% antibiotic and antimycotic (GIBCO^®^). In 96-well plates, 100 µL of cell suspension at a concentration of 106 cells/mL were placed in each well. The plates were incubated in a humidified incubator for 24 h at 37 °C under 5% CO2 atmosphere to allow cell adhesion to the plate. The cultivation medium was then replaced by 100 µL of compound solution (stocks in DMSO were diluted in cultivation medium, with final DMSO concentration no higher than 1%) in concentrations ranging from 100 to 0.39 µg·mL^−1^ and the plates were incubated for 24 h under the same conditions. Resazurin solution was added to each well and fluorescence readings (530 nm excitation, 590 nm emission) were performed after 2 h incubation. Metabolic reduction of resazurin to resorufin was used as indicator of cell viability and IC50 was defined as the highest compound concentration at which 50% of the cells were viable relative to the negative control [28]. A doxorubicin (Fauldoxo^®^) control was used for comparison; cell growth in cultivation medium and sterile medium were used respectively as positive and negative controls.

## 4. Conclusions

We have shown that 1,2,4-benzenetriol (BTO) is a suitable precursor for the synthesis of a series of new compounds that are active against citrus canker. This was done by the synthesis of alkyl derivatives (alkyl-BDOs) via a three-step protocol which showed bactericidal activity against *X. citri.* The main economic and environmental advantages of using BTO as starting material for the synthesis of new compounds are that it can be obtained from low-cost raw material while providing a use for agricultural waste. However, the synthesis route by which it was possible to obtain the compounds described here does not conform to the principles of green chemistry and needs to be improved by selective alkylation or esterification procedures. Alkyl-BDOs showed bactericidal activity against Gram-positive (*B. subtilis* and *L. lactis*) and negative (*X. citri*) bacteria, acting through the permeabilization of their cell membranes. The obtained alkyl-BDOs had a performance similar to that of gallates and dihydroxybenzoates (with greener synthesis route) previously studied by our group, and therefore, it seems to us that the disadvantages caused by the synthesis of alkyl-BDOs do not outweigh the possible advantages of using BTO as a starting material. Nevertheless, we consider that 1,2,4-benzenetriol is certainly a molecule of interest as a precursor for the synthesis of new compounds against citrus canker in the future, especially if HMF, and thus BTO, become general cheap, readily available bio-based building blocks.

## Figures and Tables

**Table 1:** MIC/MBC (in µg·mL^−1^) of alkyl-BDOs and two intermediate compounds. MIC: minimum inhibitory concentration; MBC: minimum bacteriostatic/bactericidal concentration; MW: Molecular Weigth; *B. subtilis*: *Bacillus subtilis*; *E. coli*: *Escherichia coli; L. lactis*: *Lactococcus lactis.*

	Compound	R	MW	*X. citri*	*B. subtilis*	*E. coli*	*L. lactis*
	4-BDO	(CH_2_)_3_CH_3_	182.22	50	>100	>100	100
**alkyl-BDOs**	5-BDO	(CH_2_)_4_CH_3_	196.25	50	100	>100	100
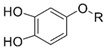	6-BDO	(CH_2_)_5_CH_3_	210.27	50	50	>100	50
	7-BDO	(CH_2_)_6_CH_3_	224.30	50	50	>100	25
	8-BDO	(CH_2_)_7_CH_3_	238.33	100	50	>100	25
	9-BDO	(CH_2_)_8_CH_3_	252.35	>100	100	>100	100
	12-BDO	(CH_2_)_11_CH_3_	294.44	>100	>100	>100	100
	14-BDO	(CH_2_)_13_CH_3_	322.49	>100	>100	>100	>100
**Intermediates**							
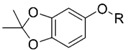	pBTO	H	166.18	>100	>100	>100	>100
	p7-BDO	(CH_2_)_6_CH_3_	264.37	>100	>100	>100	>100

**Table 2:** Percentage of *X. citri* and *B. subtilis* cells with intact or permeabilized membrane after treatment with intermediate p7-BDO and compound 7-BDO observed by fluorescence microscopy imaging.

Treatment	*X. citri*		*B. subtilis*	
	Intact	Permeabilized	Intact	Permeabilized
p7-BDO	69	31	94	6
7-BDO	0	100	0	100
negative	93	7	86	15
positive	0	100	0	100

*n* > 225 cells per treatment.

**Table 3:** Comparison of MIC (in µg·mL^−1^) of alkyl-BDOs and two intermediates with and without compounds’ pre-incubation.

Compound	*X. citri*		*B. subtilis*	
Pre-Incubation	No	24 h	No	24 h
4-BDO	50	50	>100	>100
5-BDO	50	50	100	>100
6-BDO	50	50	50	>100
7-BDO	50	50	50	>100
8-BDO	100	100	50	>100
9-BDO	>100	>100	100	>100
pBTO	>100	>100	>100	>100
p7-BDO	>100	>100	>100	>100

**Table 4:** Comparison of MIC (in µg·mL^−1^) of alkyl-BDOs and two intermediates against *X. citri* in growth media with different composition, with and without pre-incubation of the compounds. NYG: nitrogen–yeast–glycerol medium; LB: Lysogeny broth.

*X. citri* MIC/MBC				
Compound	NYG	NYG	LB–Lennox	LB–Lennox
Pre-Incubation	No	24 h	No	24 h
4-BDO	50	50	50	100
5-BDO	50	50	50	100
6-BDO	50	50	50	100
7-BDO	50	50	50	>100
8-BDO	100	100	100	>100
9-BDO	100	100	100	>100
pBTO	>100	>100	>100	>100
p7-BDO	>100	>100	>100	>100

**Table 5:** Cytotoxicity index (IC50) of compounds 6-BDO, 7-BDO, and 8-BDO against cell lines MRC-5 and J774A.1.

Compound	IC_50_ *	
	MRC-5	J774A.1
6-BDO	38.00 ± 4	>100
7-BDO	34.41 ± 4	>100
8-BDO	39.33 ± 5	>100
doxorubicin	8.7	-

* values expressed in µg·mL^−1.^

**Table 6:** Bacterial strains and standard growth conditions.

Strain	Growth Conditions *
*Xanthomonas citri* subsp. *citri* 306	NYGB (30 °C)
*Bacillus subtilis* 168	LB–Lennox (30 °C)
*Escherichia coli* MG1655	LB–Lennox (30 °C)
*Lactococcus lactis* MG1363	M17 + glucose 0.5% (30 °C)

* NYGB: peptone 5 g·L^−1^, yeast extract 3 g·L^−1^, glycerol 20 g·L^−1^ (pH ~6.7). LB–Lennox: tryptone 10 g·L^−1^, yeast extract 5 g·L^−1^, NaCl 5 g·L^−1^ (pH ~7). M17: tryptone 5 g·L^−1^, peptone 5 g·L^−1^, yeast extract 5 g·L^−1^, beef extract 2.5 g·L^−1^, ascorbic acid 0.5 g·L^−1^, MgSO4 0.25 g·L^−1^, disodium β-glycerophosphate 19 g·L^−1^ (pH ~7).

## Data Availability

Not applicable.

## Supplementary Materials

The following are available online. Section S1: NMR spectra and othercompound characterization; Section S2: Bacterial growth inhibition assessment.

## Sample Availability

Samples of the compounds described in this manuscript are available from the authors.

## Abbreviations:

alkyl-BDOs	4-alkoxy-1,2-benzene diols
BTO	1,2,4-benzenetriol
HMF	hydroxymethylfurfural
DBU	1,8-Diazabicyclo[5.4.0]undec-7-ene
pBTO	Protected BTO; 2,2-dimethylbenzo[d][1,3]dioxol-5-ol
p7-BDO	Protected intermediate; 2,2-dimethyl-5-(heptyloxy)benzo[d][1,3]dioxole
4-BDO	4-(butoxy)benzene-1,2-diol
5-BDO	4-(pentyloxy)benzene-1,2-diol
6-BDO	4-(hexyloxy)benzene-1,2-diol
7-BDO	4-(heptyloxy)benzene-1,2-diol
8-BDO	4-(octyloxy)benzene-1,2-diol
9-BDO	4-(nonyloxy)benzene-1,2-diol
12-BDO	4-(dodecyloxy)benzene-1,2-diol
14-BDO	4-(tetradecyloxy)benzene-1,2-diol
*X. citri*	*Xanthomonas citri* subsp. *citri*
*B. subtilis*	*Bacillus subtilis*
*E. coli*	*Escherichia coli*
*L. lactis*	*Lactococcus lactis*
MICs	Minimum inhibitory concentrations
MBC	Minimum bacteriostatic/Bactericidal concentration
DMSO	Dimethyl sulfoxide
NYG	Nitrogen–yeast–glycerol medium
LB–Lennox	Lysogeny broth–Lennox medium
IC50	Cytotoxicity index
EtOAc	Ethyl acetate
NMR	Nuclear magnetic resonance
HRMS	High-resolution mass spectrometry
FTIR	Fourier-transform infrared spectroscopy
OD600	Optical density at 600 nm
