# Supplementary material for: Benzenetriol-Derived Compounds against Citrus Canker
Source: Molecules. 2021 Mar 6;26(5):1436. doi: 10.3390/molecules26051436 (PMC7961829; doi:10.3390/molecules26051436)
Supplement: Supplementary file 1 [file molecules-26-01436-s001.pdf]

molecules

MDPI

SUPPORTING INFORMATION

Benzenetriol-derived compounds against citrus canker

Lúcia Bonci Cavalca [1,2], Ciaran W. Lahive [2], Fleur Gijsbers [1], Fernando Rogério Pavan [3], Dirk-Jan Scheffers [1,]* and Peter J. Deuss [2,]*

[1] Department of Molecular Microbiology, Groningen Biomolecular Sciences and Biotechnology Institute, University of Groningen, Groningen, The Netherlands.

[2] Department of Chemical Engineering (ENTEG), University of Groningen, Nijenborgh 4, 9747 AG Groningen, The Netherlands.

[3] School of Pharmaceutical Sciences, São Paulo State University (UNESP), Araraquara, Brazil.

* Correspondence: d.j.scheffers@rug.nl; p.j.deuss@rug.nl

Section S1 NMR spectra and compounds' characterization

Table S1: Alkyl-BDOs (BDOs) and intermediates.

	compound	R	MW	yield			
				mg	mmol	over 2 steps (%)	over 3 steps (%)
**alkyl-BDO** 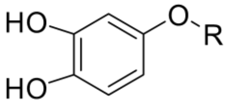	4-BDO	$(CH_2)_3CH_3$	182.22	126	0.69	29	9
	5-BDO	$(CH_2)_4CH_3$	196.25	108	0.55	23	7
	6-BDO	$(CH_2)_5CH_3$	210.27	144	0.68	28	9
	7-BDO	$(CH_2)_6CH_3$	224.30	100	0.45	19	6
	8-BDO	$(CH_2)_7CH_3$	238.33	169	0.71	30	9
	9-BDO	$(CH_2)_8CH_3$	252.35	123	0.49	20	6
	12-BDO	$(CH_2)_{11}CH_3$	294.44	-			
	14-BDO	$(CH_2)_{13}CH_3$	322.49	-			
**intermediates**	pBTO	H	166.18	400	2.41*		
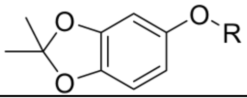	p-BDOs	$(CH_2)_{3-13}CH_3$	-	417-500	0.5 - 0.7		

Starting material: 1g BTO; - not determined; * pBTO yield (first step): 30%

Final products:

4-(butoxy)benzene-1,2-diol (4-BDO)

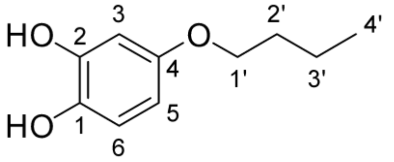

white solid: melting point 93-94°C (Discoloration was observed before the melting point was reached). **^{1}H NMR (400 MHz, DMSO-*d*$_6$)** δ 8.78 (s, 1H, OH), 8.35 (s, 1H, OH), 6.59 (d, *J* = 8.6 Hz, 1H, Ar), 6.32 (d, *J* = 2.9 Hz, 1H, Ar), 6.17 (dd, *J* = 8.6, 2.9 Hz, 1H, Ar), 3.79 (t, *J* = 6.5 Hz, 2H, C1′), 1.69 – 1.58 (m, 2H, C2′), 1.30 – 1.22 (m, 2H, C3′), 0.94 – 0.88 (m, 3H, C4′). **^{13}C NMR (100 MHz, DMSO-*d*$_6$)** δ 151.97, 145.84, 139.02, 115.63, 104.21, 103.16, 67.30 (C1′), 30.91, 18.77, 13.71 (C4′). **HRMS** (ESI) calculated for $C_{10}H_{13}O_3^-$ ([M-H]$^-$): 181.08702, found 181.08725.

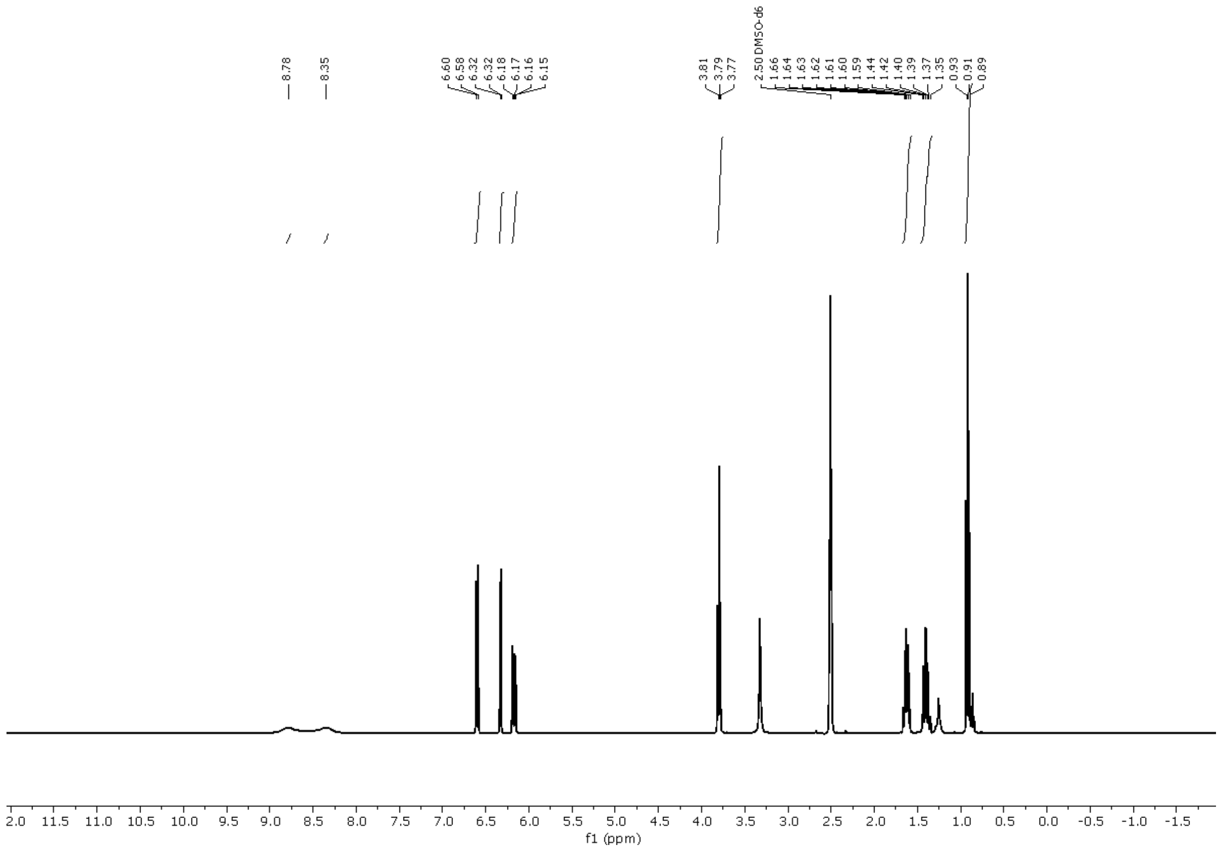

Figure S1.1a: ^{1}H NMR (400 MHz, DMSO-*d*$_6$) spectrum of compound 4-BDO.

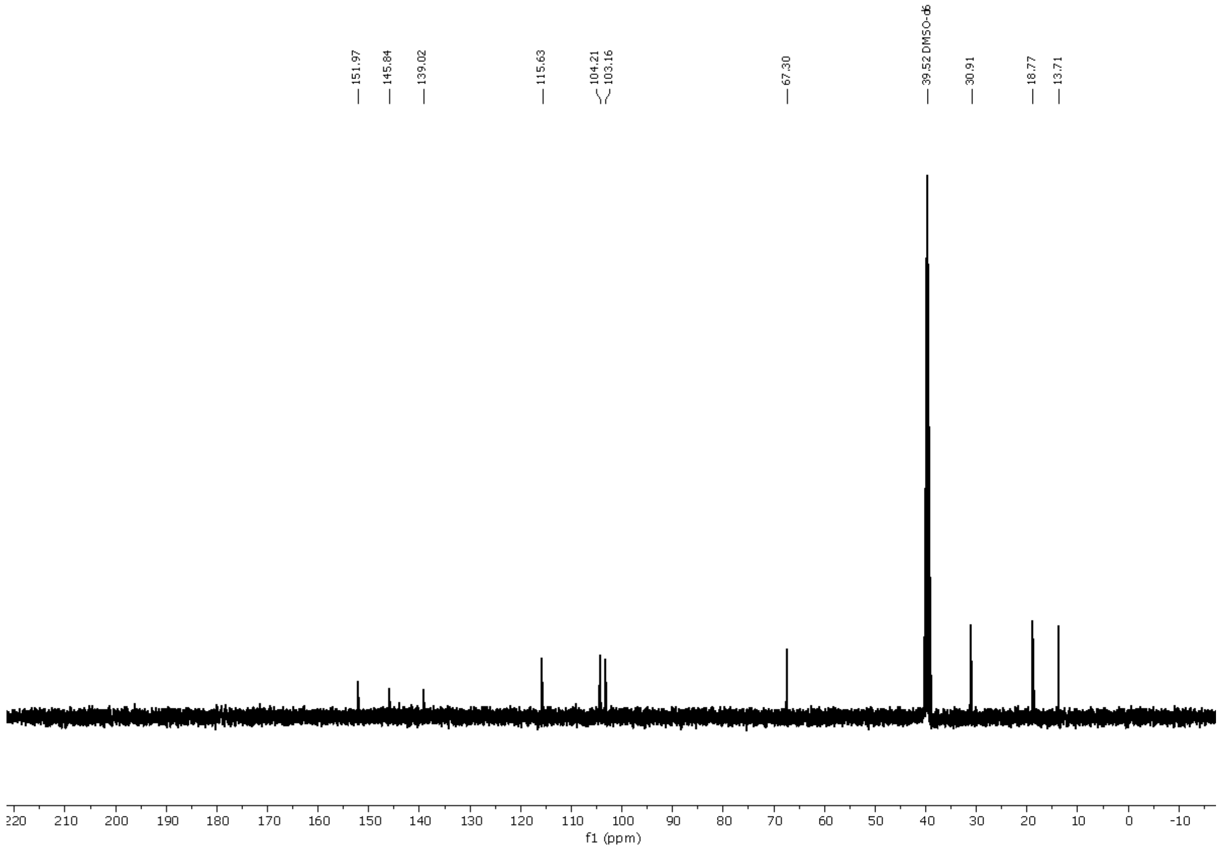

Figure S1.1b: ^{13}C NMR (100 MHz, DMSO-*d*$_6$) spectrum of compound 4-BDO.

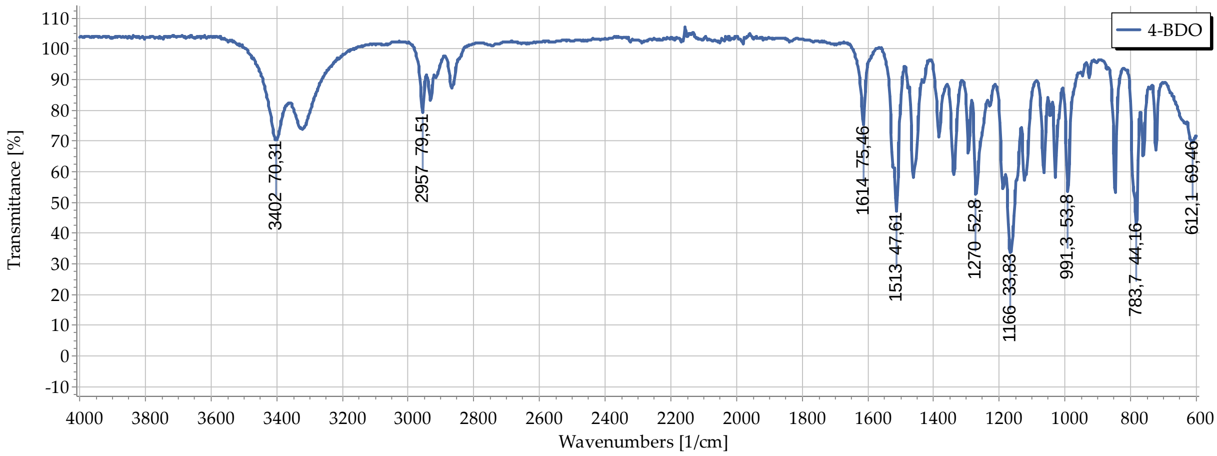

Figure S1.1c: FTIR spectrum of compound 4-BDO.

4-(pentyloxy)benzene-1,2-diol (5-BDO)

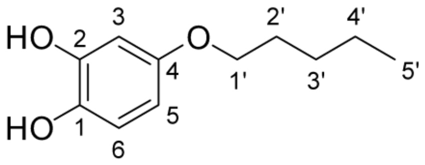

white solid: ^{1}**H NMR (400 MHz, DMSO-*d*$_6$)** δ 8.81 (s, 1H, OH), 8.31 (s, 1H, OH), 6.59 (d, *J* = 8.6 Hz, 1H, Ar), 6.32 (d, *J* = 2.9 Hz, 1H, Ar), 6.16 (dd, *J* = 8.6, 2.9 Hz, 1H, Ar), 3.78 (t, *J* = 6.5 Hz, 2H, C1'), 1.69 – 1.58 (m, 2H, C2'), 1.42 – 1.25 (m, 4H, C3'- C4'), 0.94 – 0.84 (m, 3H, C5'). ^{13}**C NMR (100 MHz, DMSO-*d*$_6$)** δ 151.98, 145.86, 139.05, 115.65, 104.22, 103.18, 67.61 (C1'), 28.54, 27.78, 21.92, 13.93 (C5'). **HRMS** (ESI) calculated for $C_{11}H_{15}O_3^-$ ([M-H]$^-$): 195.10267, found 195.10274.

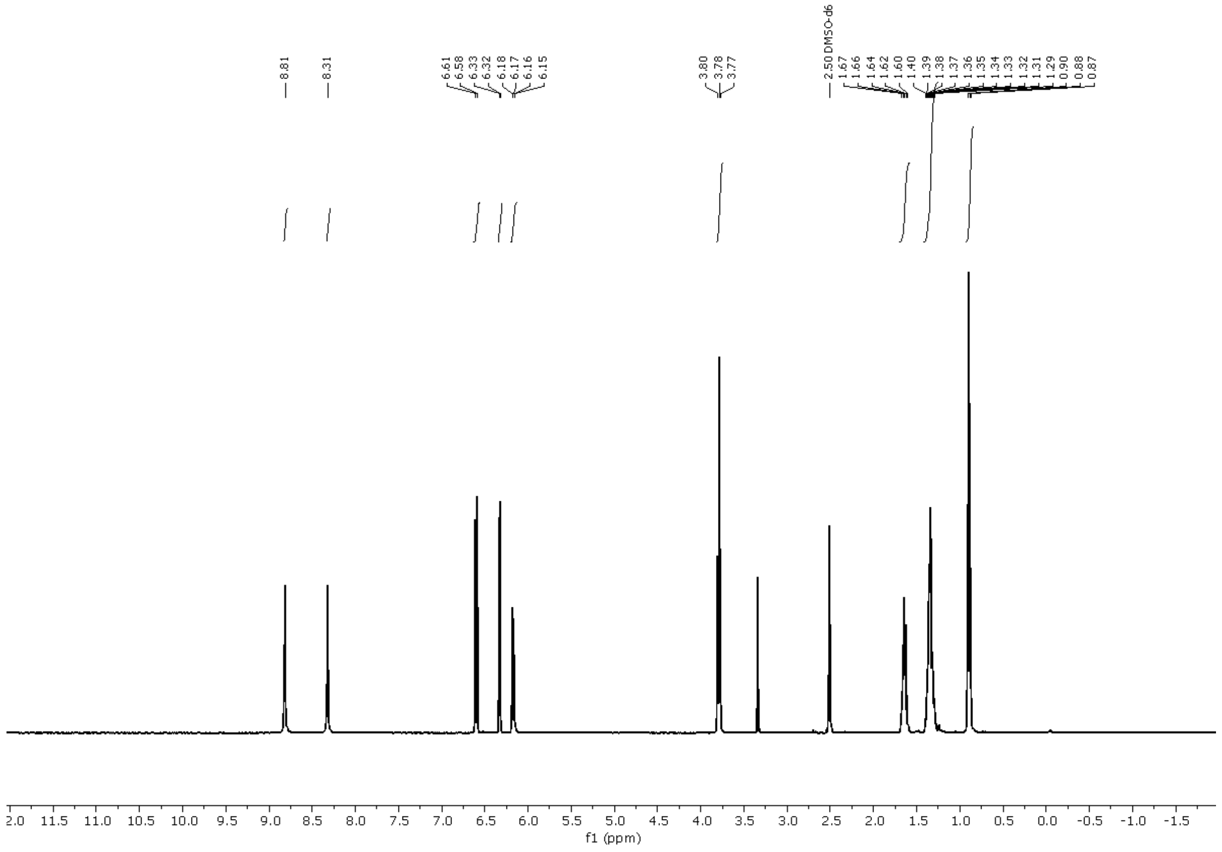

Figure S1.2a: ^{1}H NMR (400 MHz, DMSO-d_6) spectrum of compound 5-BDO.

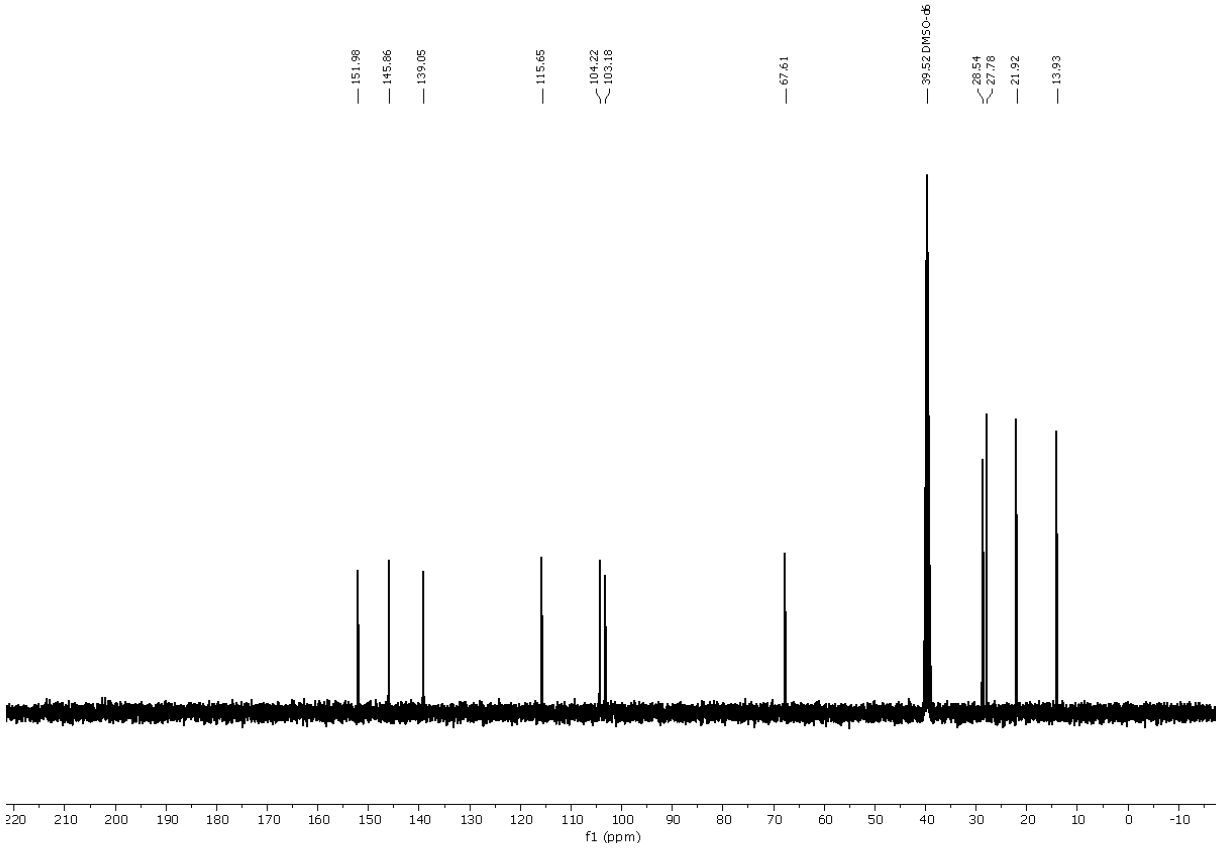

Figure S1.2b: ^{13}C NMR (100 MHz, DMSO-d_6) spectrum of compound 5-BDO.

4-(hexyloxy)benzene-1,2-diol (6-BDO)

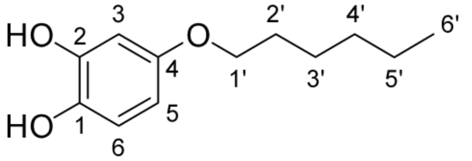

white solid: **^{1}H NMR (400 MHz, DMSO-*d*$_6$)** δ 8.81 (s, 1H, OH), 8.31 (s, 1H, OH), 6.59 (d, *J* = 8.6 Hz, 1H, C5), 6.32 (d, *J* = 2.9 Hz, 1H, C2), 6.16 (dd, *J* = 8.6, 2.9 Hz, 1H, C6), 3.78 (t, *J* = 6.5 Hz, 2H, C1'), 1.69 – 1.58 (m, 2H, C2'), 1.43 – 1.33 (m, 2H, C3'), 1.33 - 1.22 (m, 4H, C4'- C5'), 0.92 – 0.82 (m, 3H, C6'). **^{13}C NMR (100 MHz, DMSO-*d*$_6$)** δ 151.98, 145.86, 139.05, 115.65, 104.22, 103.18, 67.62 (C1'), 31.05, 28.81, 25.25, 22.08, 13.91 (C6'). **HRMS** (ESI) calculated for $C_{12}H_{17}O_3^-$ ([M-H]$^-$): 209.11832, found 209.11815.

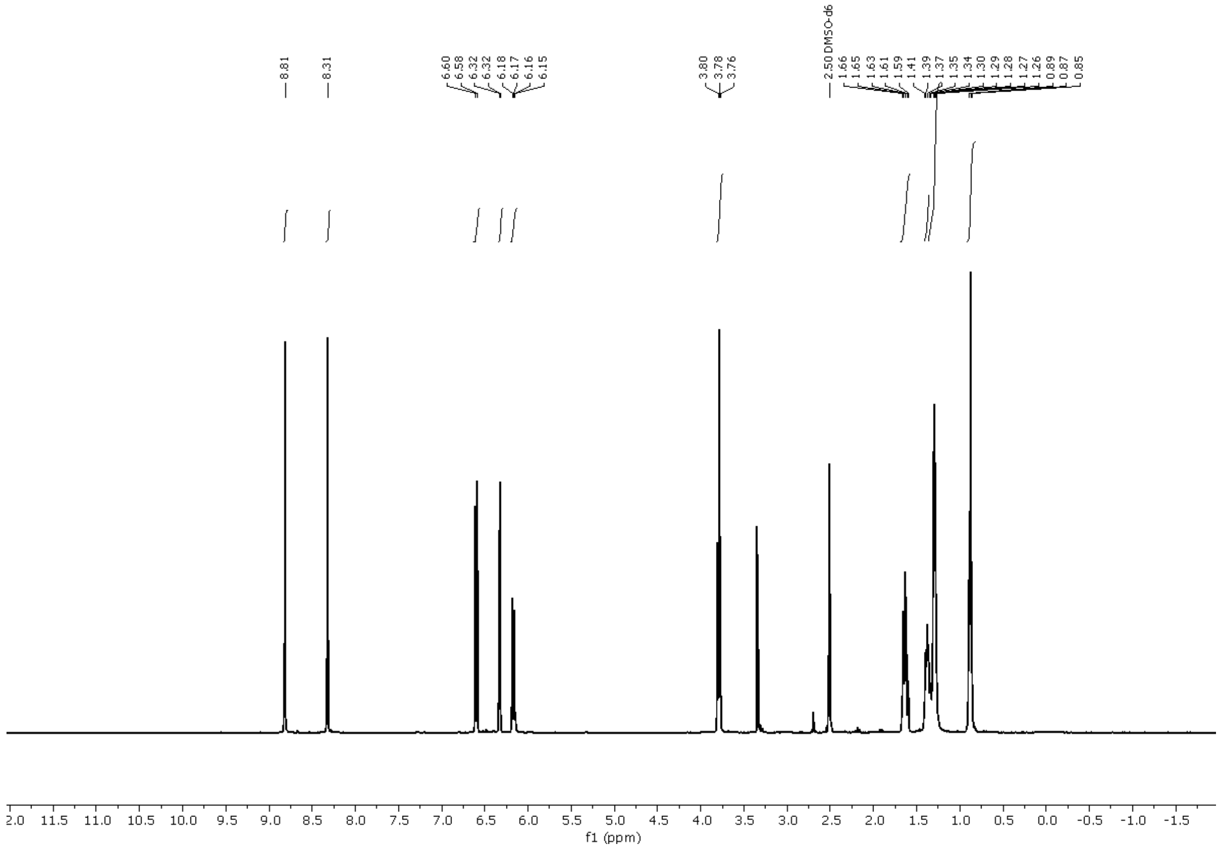

Figure S1.3a: ^{1}H NMR (400 MHz, DMSO-*d*$_6$) spectrum of compound 6-BDO.

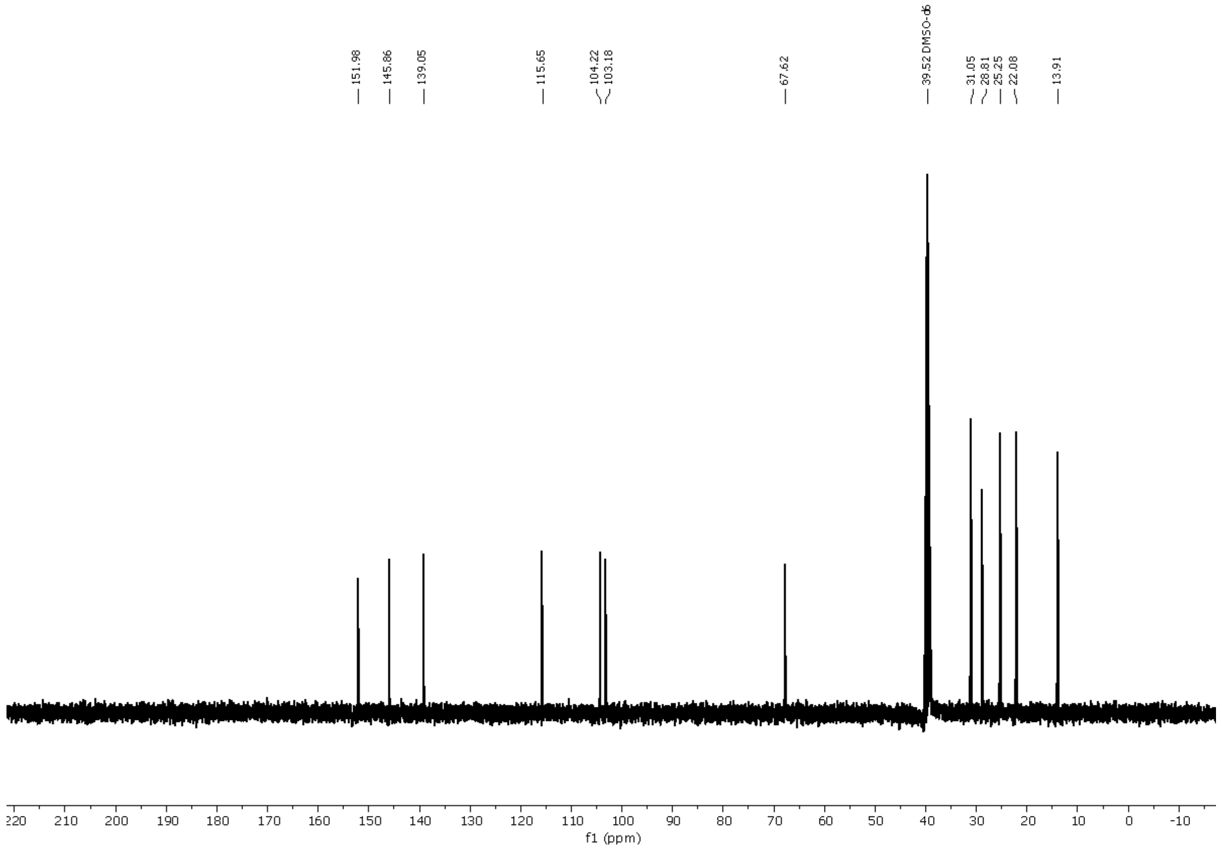

Figure S1.3b: ^{13}C NMR (100 MHz, DMSO-d_6) spectrum of compound 6-BDO.

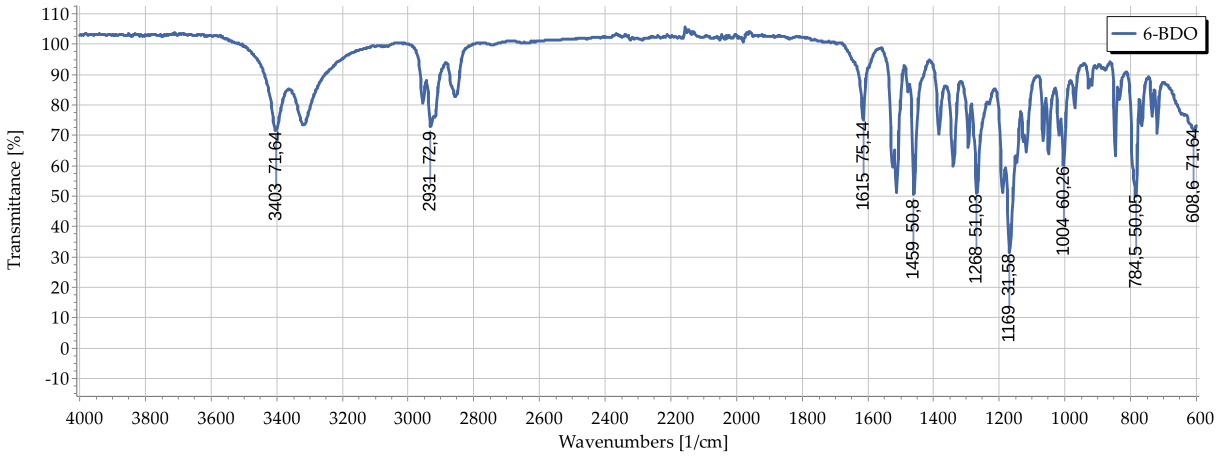

Figure S1.3c: FTIR spectrum of compound 6-BDO.

4-(heptyloxy)benzene-1,2-diol (7-BDO)

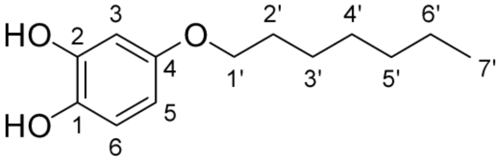

white solid: **^{1}H NMR (400 MHz, DMSO-d_6)** δ 8.81 (s, 1H, OH), 8.31 (s, 1H, OH), 6.59 (d, *J* = 8.6 Hz, 1H, C5), 6.32 (d, *J* = 2.9 Hz, 1H, C2), 6.16 (dd, *J* = 8.6, 2.9 Hz, 1H, C6), 3.78 (t, *J* = 6.5 Hz, 2H, C1′), 1.69 – 1.58 (m, 2H, C2′), 1.42 – 1.32 (m, 2H, C3′), 1.32 - 1.22 (m, 6H, C4′- C6′), 0.90 – 0.82 (m, 3H, C7′). **^{13}C NMR (100 MHz,**

DMSO-*d*6) δ 151.97, 145.85, 139.04, 115.64, 104.21, 103.17, 67.61 (C1′), 31.26, 28.85, 28.48, 25.54, 22.06, 13.94 (C7′). **HRMS** (ESI) calculated for $C_{13}H_{19}O_3^-$ ([M-H]$^-$): 223.13397, found 223.13404.

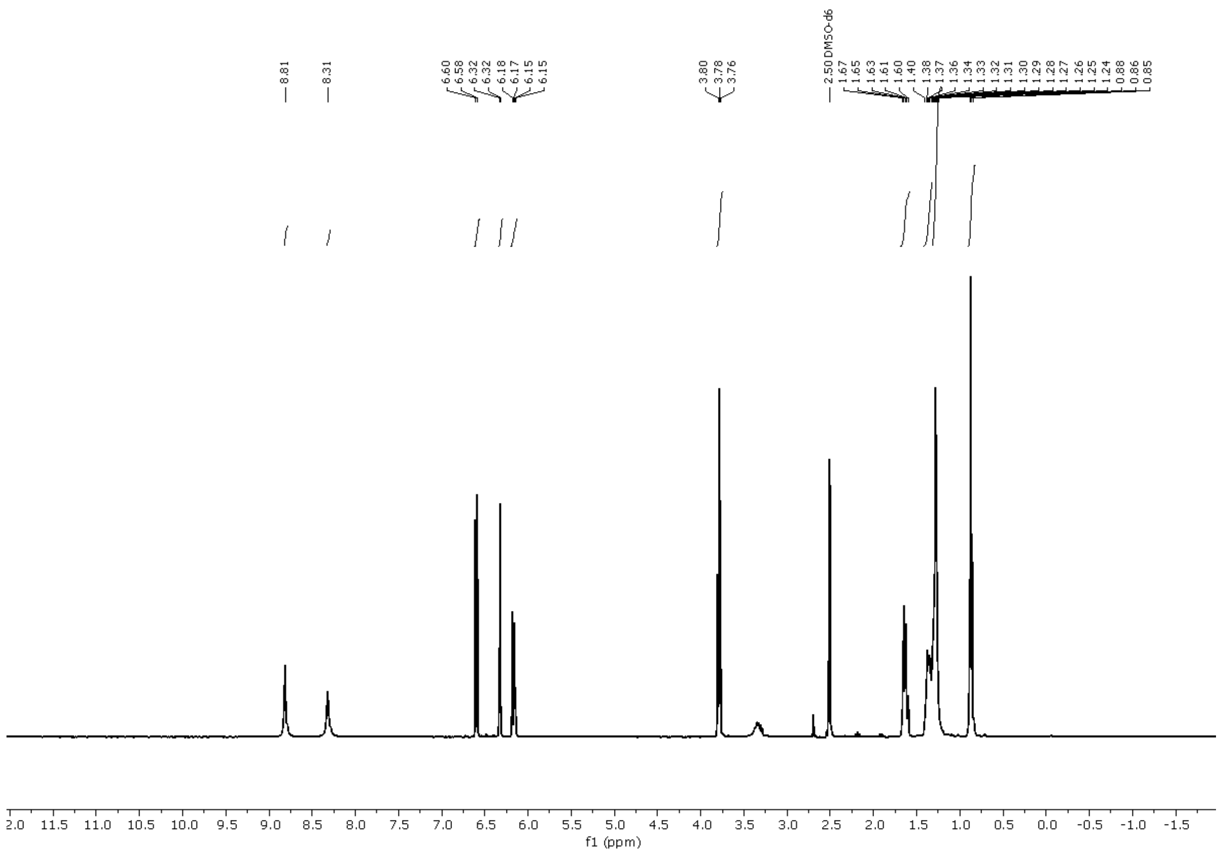

Figure S1.4a: ^{1}H NMR (400 MHz, DMSO-*d*6) spectrum of compound 7-BDO.

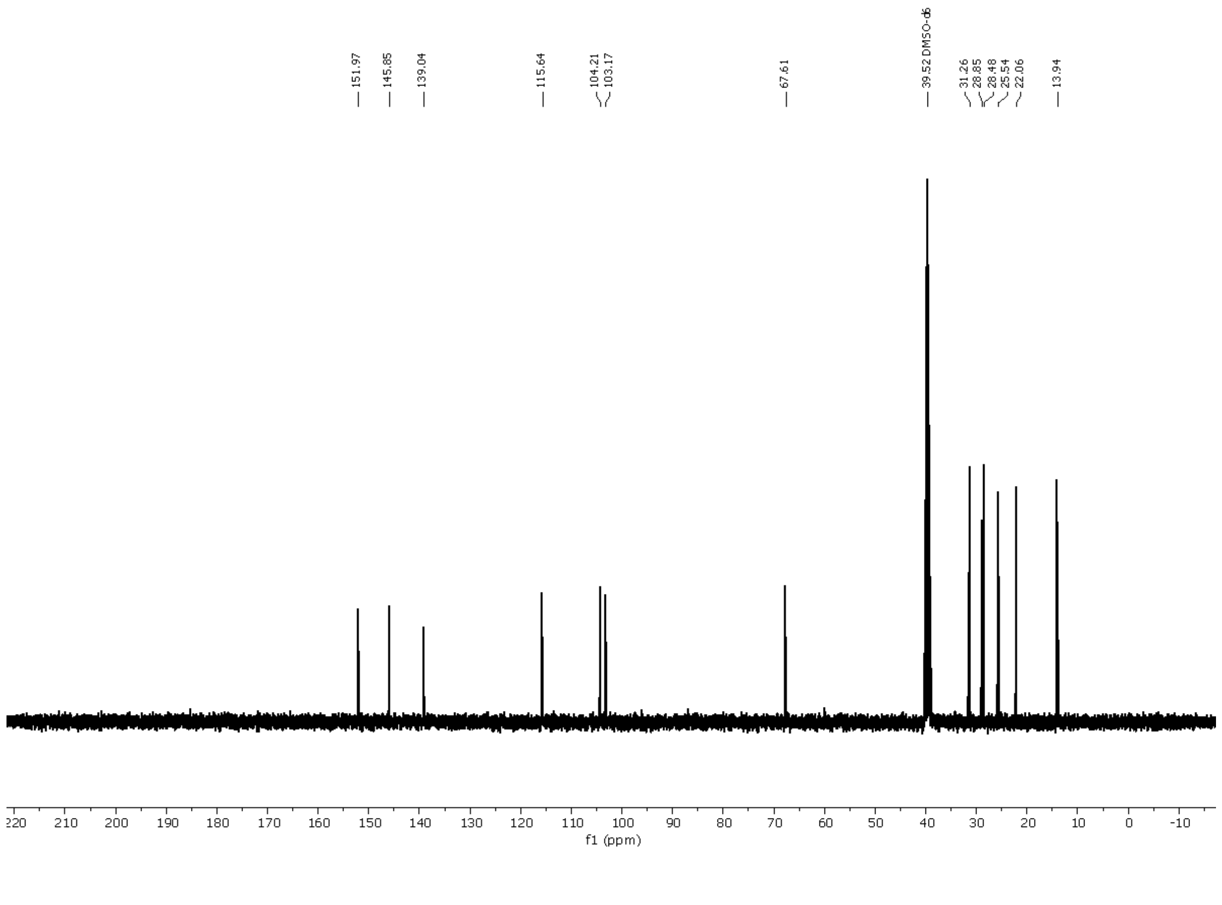

Figure S1.4b: ^{13}C NMR (100 MHz, DMSO-d_6) spectrum of compound 7-BDO.

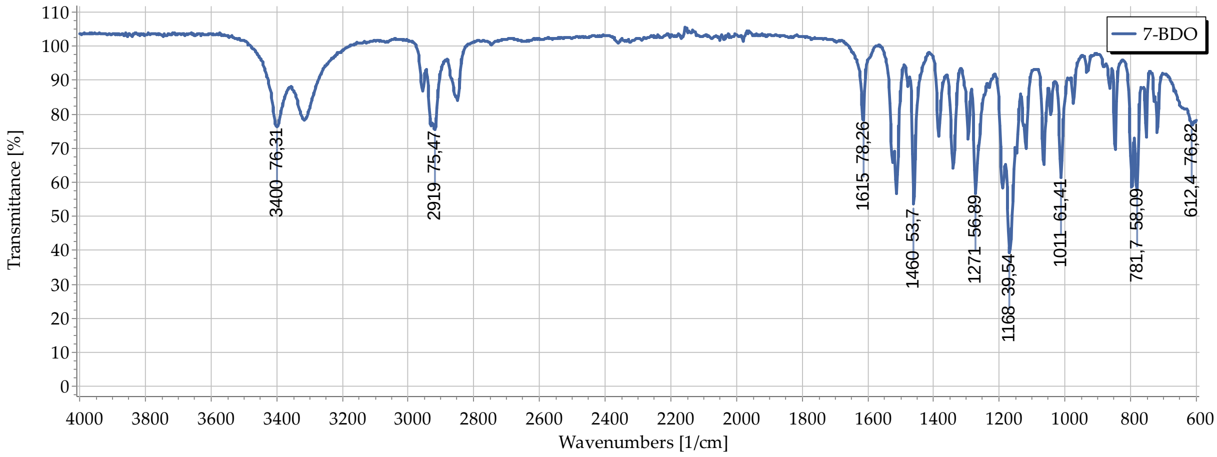

Figure S1.4c: FTIR spectrum of compound 7-BDO.

4-(octyloxy)benzene-1,2-diol (8-BDO)

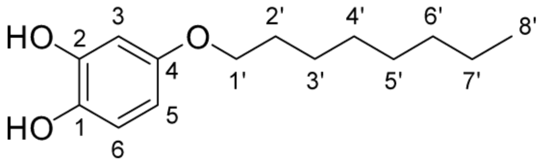

white solid: melting point 100-102 °C (Discoloration was observed before the melting point was reached). **^{1}H NMR (400 MHz, DMSO-d_6)** δ 8.81 (s, 1H, OH), 8.31 (s, 1H, OH), 6.59 (d, J = 8.6 Hz, 1H, C5), 6.32 (d, J = 2.9 Hz, 1H, C2), 6.16 (dd, J = 8.6, 2.9 Hz, 1H, C6), 3.78 (t, J = 6.5 Hz, 2H, C1′), 1.69 – 1.58 (m, 2H, C2′), 1.42 – 1.32 (m, 2H, C3′), 1.32 - 1.22 (m, 8H, C4′- C7′), 0.90 – 0.82 (m, 3H, C8′). **^{13}C NMR (100 MHz, DMSO-d_6)** δ 151.97, 145.85, 139.04, 115.63, 104.20, 103.17, 67.61 (C1′), 31.25, 28.84, 28.78, 28.68, 25.58, 22.09, 13.95 (C8′). **HRMS** (ESI) calculated for $C_{14}H_{21}O_3^-$ ([M-H]$^-$): 237.14962, found 237.17953.

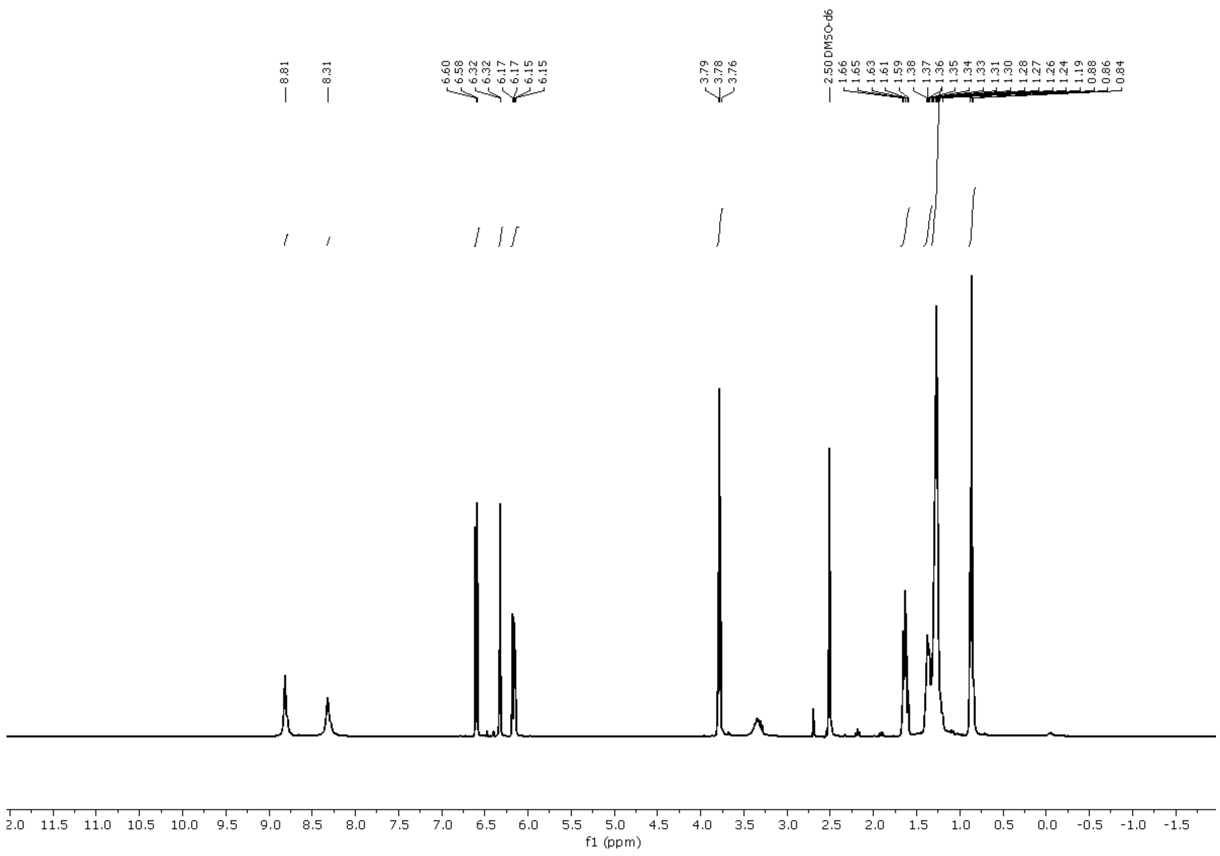

Figure S1.5a: ^{1}H NMR (400 MHz, DMSO-d_6) spectrum of compound 8-BDO.

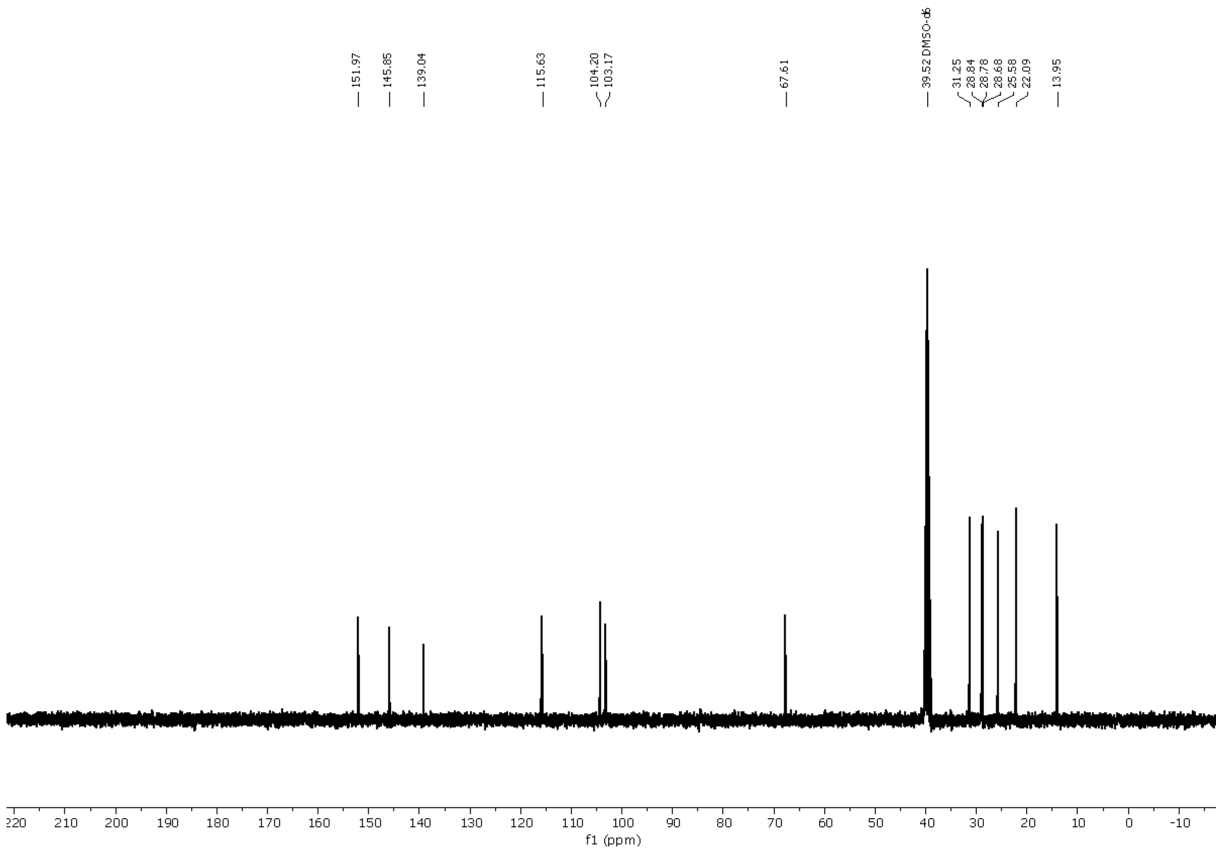

Figure S1.5b: ^{13}C NMR (100 MHz, DMSO-d_6) spectrum of compound 8-BDO.

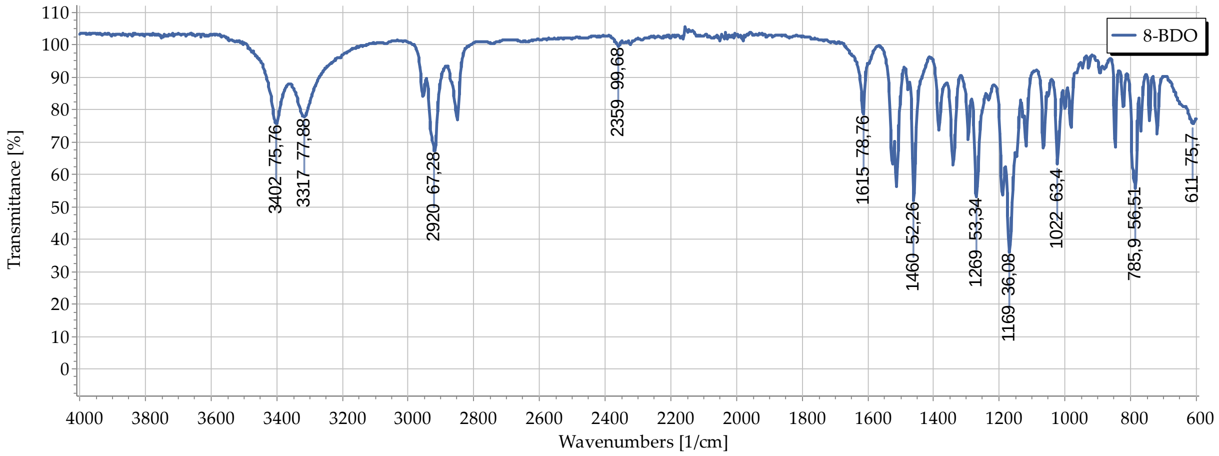

Figure S1.5c: FTIR spectrum of compound 8-BDO.

4-(nonyloxy)benzene-1,2-diol (9-BDO)

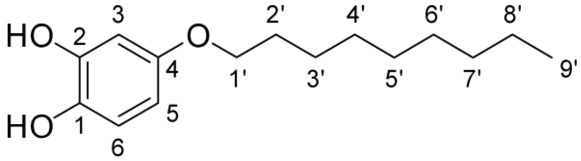

white solid: **^{1}H NMR (400 MHz, DMSO-*d*$_6$)** δ 8.80 (s, 1H, OH), 8.31 (s, 1H, OH), 6.59 (d, *J* = 8.6 Hz, 1H, C5), 6.32 (d, *J* = 2.9 Hz, 1H, C2), 6.16 (dd, *J* = 8.6, 2.9 Hz, 1H, C6), 3.78 (t, *J* = 6.5 Hz, 2H, C1′), 1.68 – 1.58 (m, 2H, C2′), 1.42 – 1.32 (m, 2H, C3′), 1.32 - 1.22 (m, 10H, C4′- C8′), 0.90 – 0.82 (m, 3H, C9′). **^{13}C NMR (100 MHz, DMSO-*d*$_6$)** δ 151.97, 145.85, 139.04, 115.63, 104.19, 103.17, 67.60 (C1′), 31.28, 28.98, 28.85, 28.82, 28.67, 25.57, 22.10, 13.95 (C9′). **HRMS** (ESI) calculated for $C_{15}H_{23}O_3^-$ ([M-H]$^-$): 251.16527, found 251.16487.

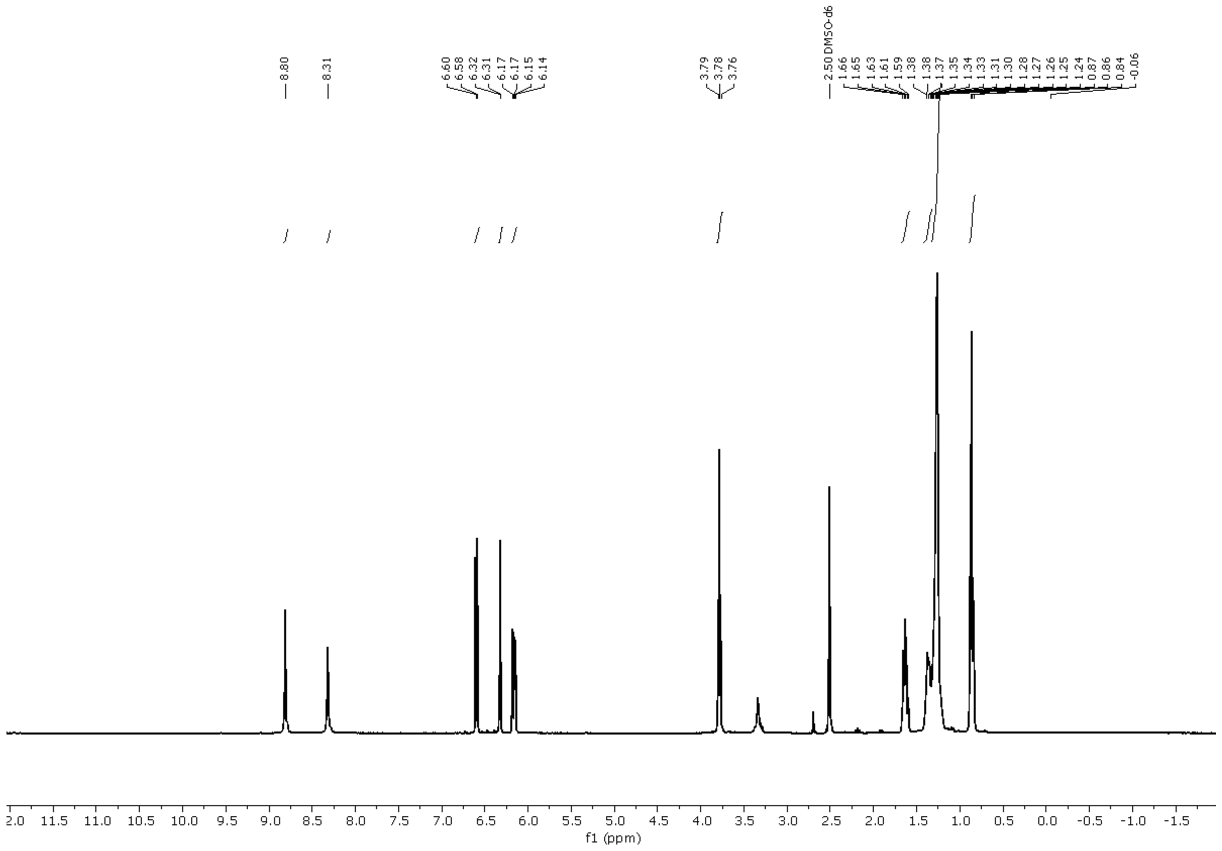

Figure S1.6a: ^{1}H NMR (400 MHz, DMSO-d_6) spectrum of compound 9-BDO.

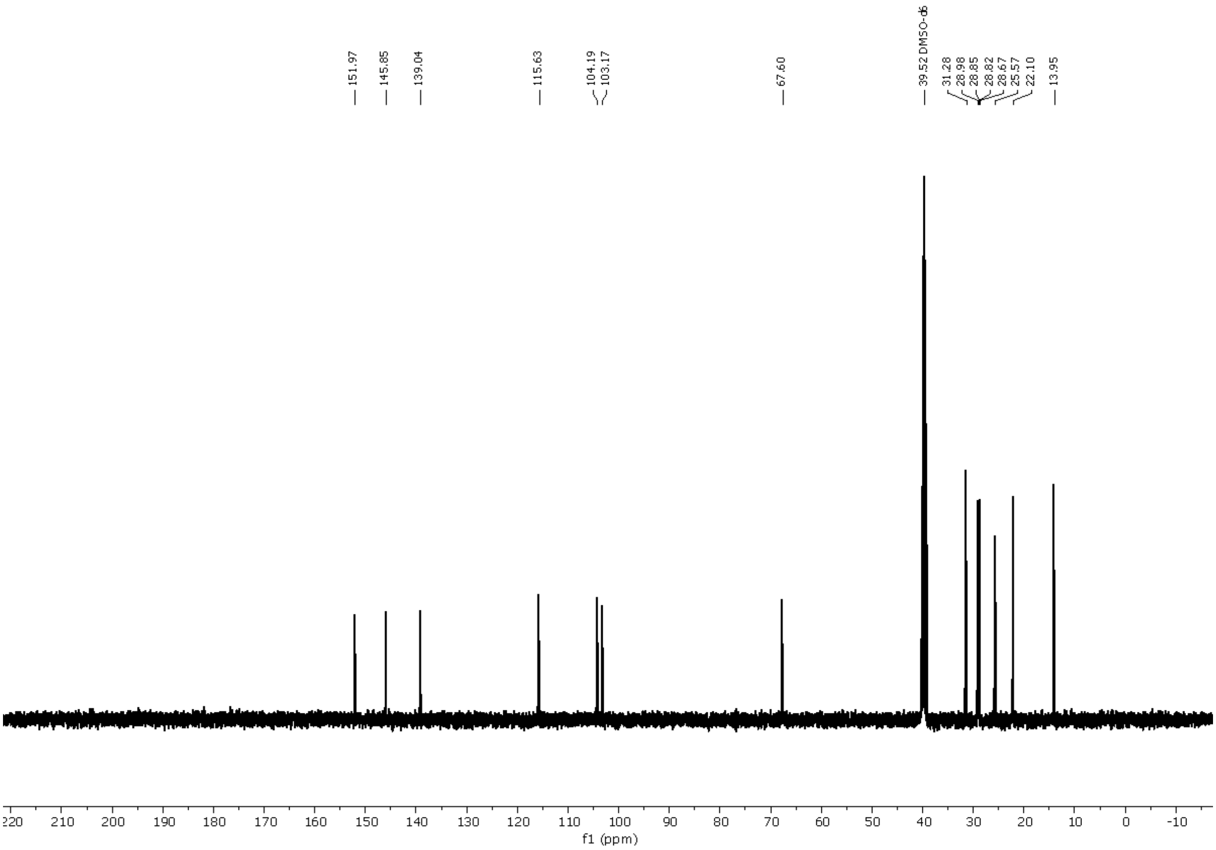

Figure S1.6b: ^{13}C NMR (100 MHz, DMSO-d_6) spectrum of compound 9-BDO.

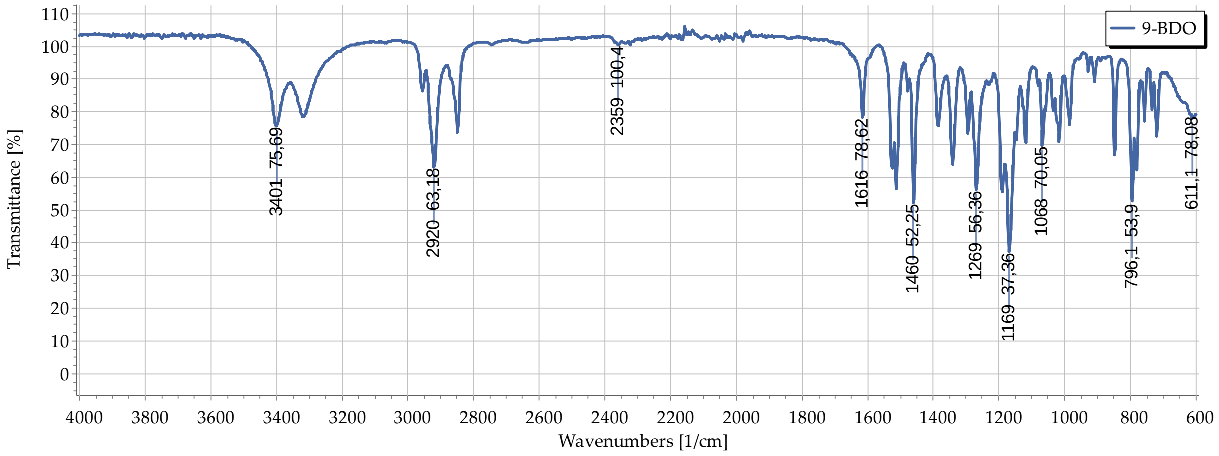

Figure S1.6c: FTIR spectrum of compound 9-BDO.

4-(dodecyloxy)benzene-1,2-diol (12C)

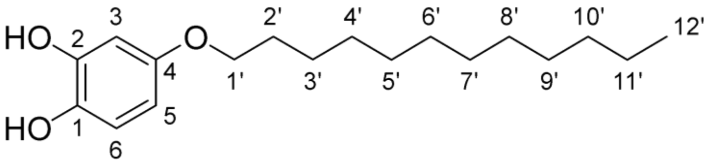

white solid: **^{1}H NMR (400 MHz, DMSO-d_6)** δ 8.80 (s, 1H, OH), 8.31 (s, 1H, OH), 6.59 (d, *J* = 8.6 Hz, 1H, C5), 6.32 (d, *J* = 2.9 Hz, 1H, C2), 6.16 (dd, *J* = 8.6, 2.9 Hz, 1H, C6), 3.77 (t, *J* = 6.5 Hz, 2H, C1'), 1.68 – 1.57 (m, 2H,

C2′), 1.40 – 1.32 (m, 2H, C3′), 1.32 - 1.22 (m, 16H, C4′- C11′), 0.89 – 0.81 (m, 3H, C12′). **^{13}C NMR (100 MHz, DMSO-*d*$_6$)** δ 151.97, 145.85, 139.04, 115.62, 104.18, 103.16, 67.60 (C1′), 31.30, 29.05, 29.02, 29.00, 28.85, 28.81, 28.72, 25.57, 22.10, 13.94 (C12′). **HRMS** (ESI) calculated for $C_{18}H_{29}O_3^-$ ([M-H]$^-$): 293.21222, found 293.21169.

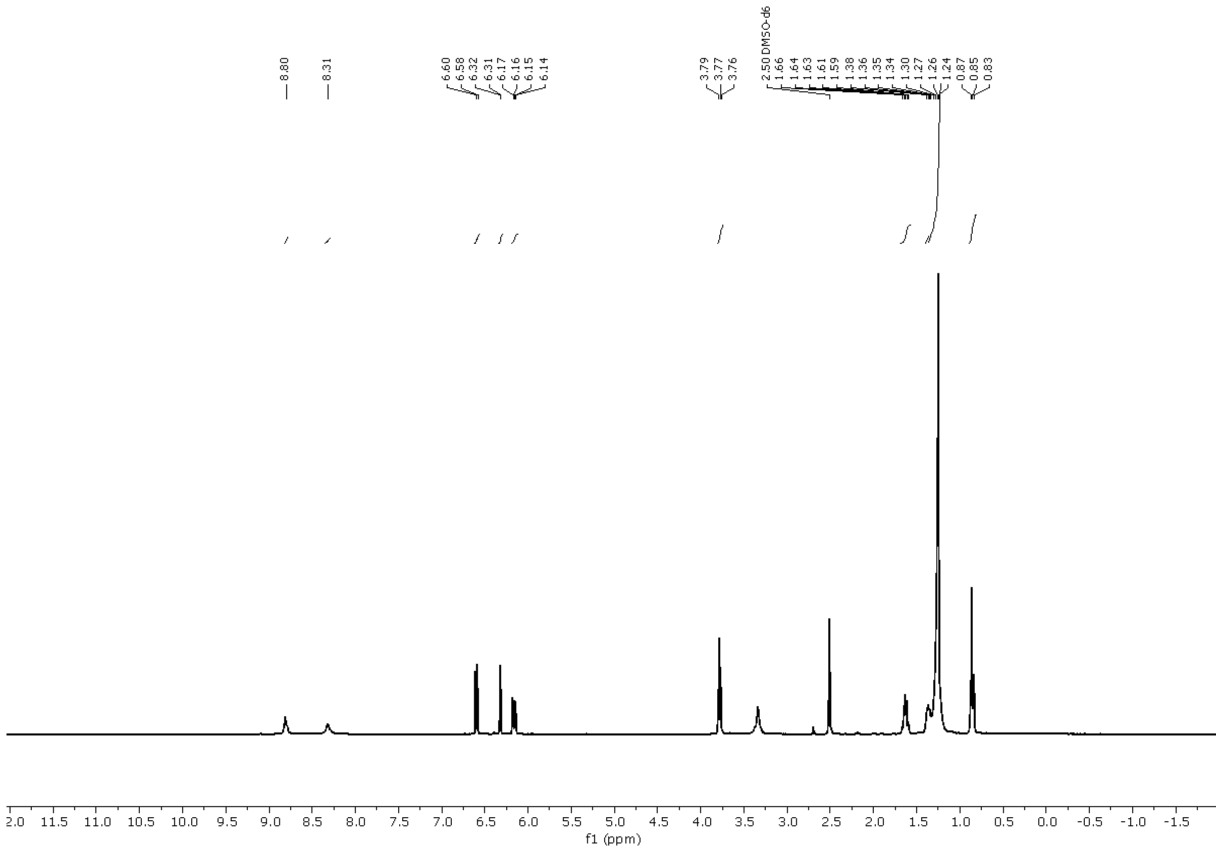

Figure S1.7a: ^{1}H NMR (400 MHz, DMSO-*d*$_6$) spectrum of compound 12C.

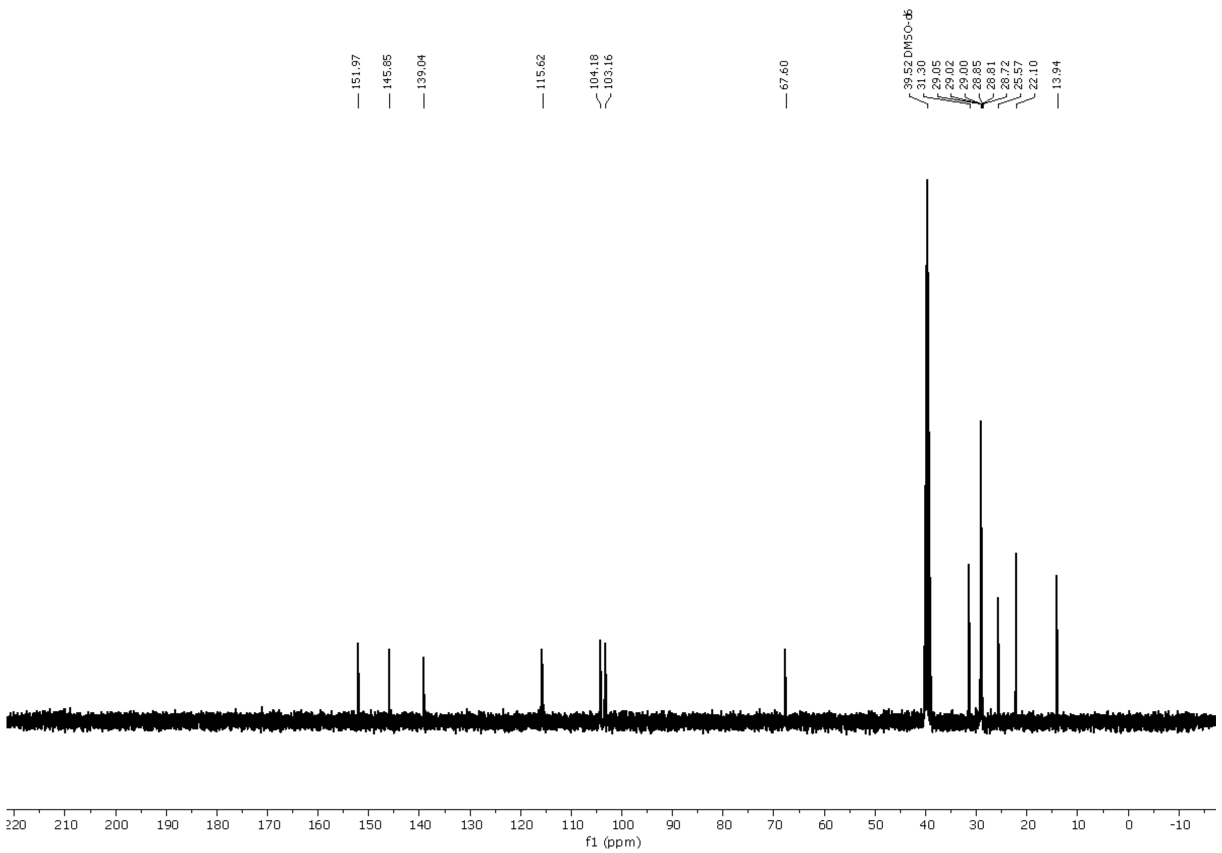

Figure S1.7b: ^{13}C NMR (100 MHz, DMSO-d_6) spectrum of compound 12C.

4-(tetradecyloxy)benzene-1,2-diol (14-BDO)

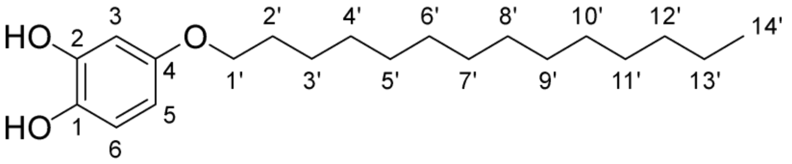

white solid: melting point 113-115 °C (Discoloration was observed before the melting point was reached). **^{1}H NMR (400 MHz, DMSO-d_6)** δ 8.80 (s, 1H, OH), 8.31 (s, 1H, OH), 6.59 (d, *J* = 8.6 Hz, 1H, C5), 6.31 (d, *J* = 2.9 Hz, 1H, C2), 6.16 (dd, *J* = 8.6, 2.9 Hz, 1H, C6), 3.77 (t, *J* = 6.5 Hz, 2H, C1′), 1.68 – 1.57 (m, 2H, C2′), 1.40 – 1.32 (m, 2H, C3′), 1.32 - 1.22 (m, 20H, C4′- C13′), 0.89 – 0.81 (m, 3H, C14′). **^{13}C NMR (100 MHz, DMSO-d_6)** δ 151.97, 145.85, 139.04, 115.61, 104.17, 103.16, 67.59 (C1′), 31.30, 29.06, 29.05, 29.04, 29.02, 29.01, 29.00, 28.85, 28.82, 28.72, 25.57, 22.10, 13.93 (C14′). **HRMS** (ESI) calculated for $C_{20}H_{33}O_3^-$ ([M-H]$^-$): 321.24352, found 321.24308.

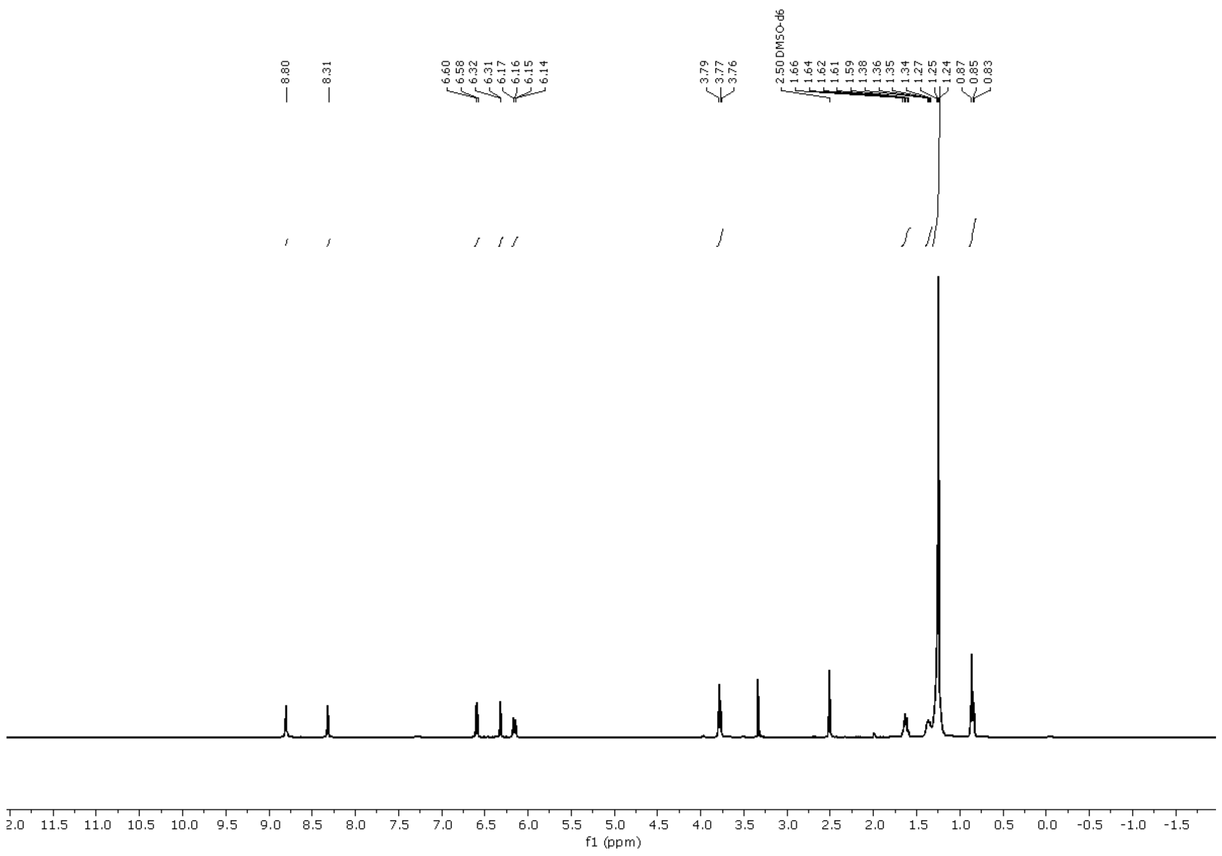

Figure S1.8a: ^{1}H NMR (400 MHz, DMSO-d_6) spectrum of compound 14-BDO.

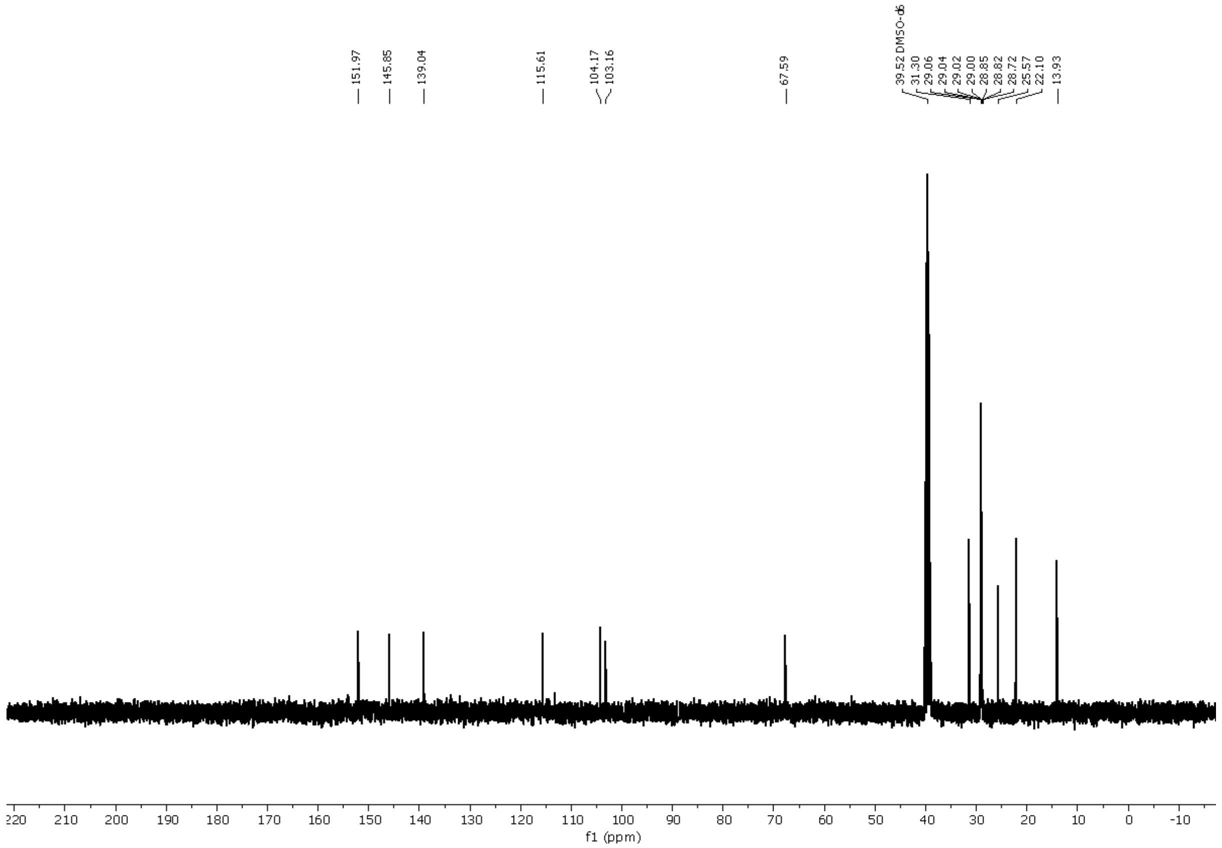

Figure S1.8b: ^{13}C NMR (100 MHz, DMSO-d_6) spectrum of compound 14-BDO.

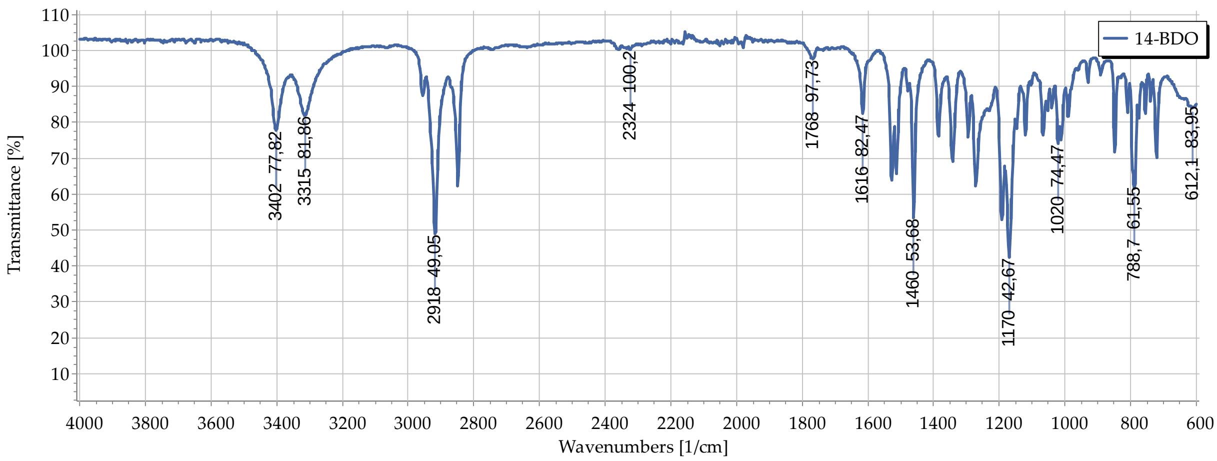

Figure S1.8c: FTIR spectrum of compound 14-BDO.

Starting material and intermediates:

Benzene-1,2,4-triol (BTO)

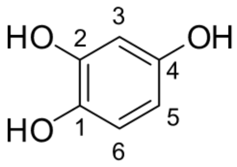

¹H NMR (400 MHz, DMSO-*d*₆) δ 8.65 (s, 1H, OH), 8.47 (s, 1H, OH), 8.2 (s, 1H, OH), 6.49 (d, *J* = 8.4 Hz, 1H, Ar), 6.21 (d, *J* = 2.8 Hz, 1H, Ar), 6.00 (dd, *J* = 8.4, 2.8 Hz, 1H, Ar).

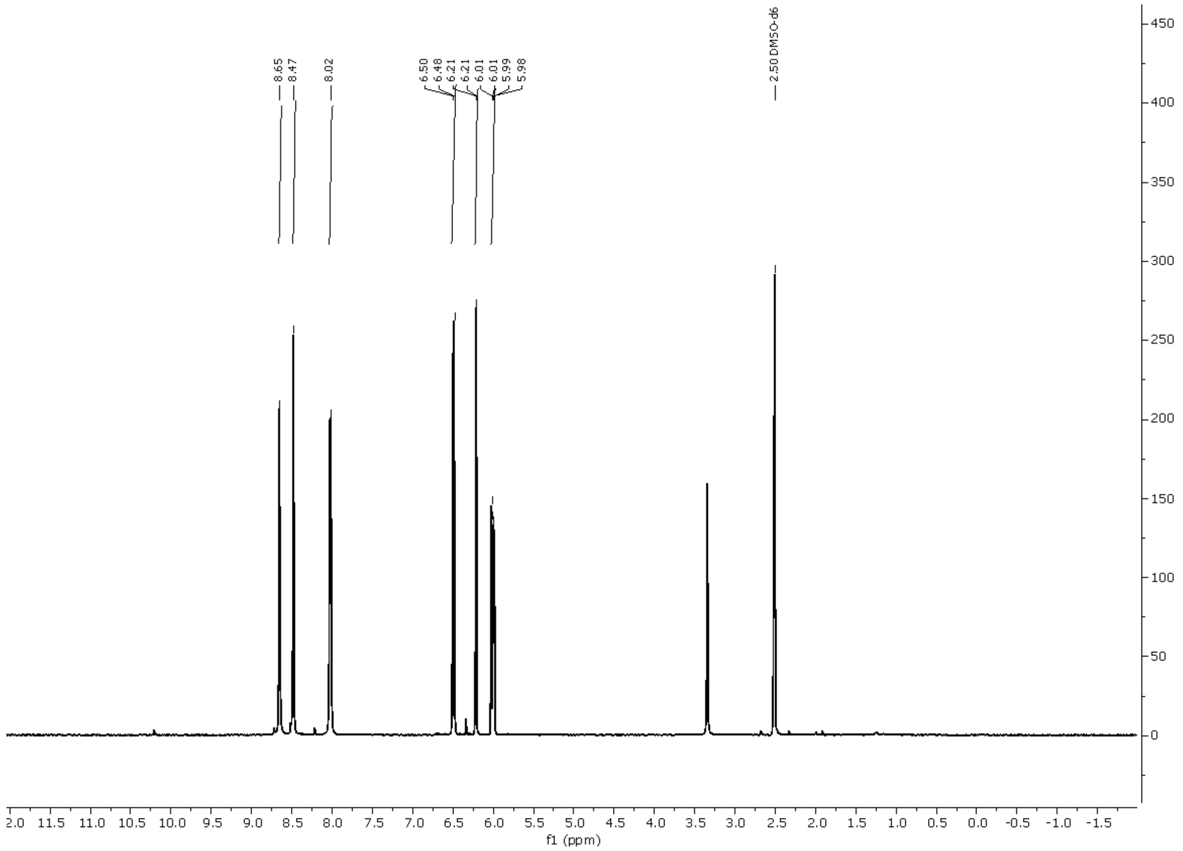

Figure S1.11: ¹H NMR (400 MHz, DMSO-*d*₆) spectrum of BTO.

2,2-dimethylbenzo[d][1,3]dioxol-5-ol (pBTO)

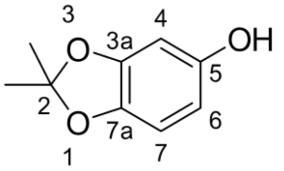

dark brown liquid: **^{1}H NMR (400 MHz, Chloroform-*d*)** δ 6.55 (d, J = 8.3 Hz, 1H, Ar), 6.34 (d, J = 2.2 Hz, 1H, Ar), 6.20 (dd, J = 8.3, 2.3 Hz, 1H, Ar), 4.66 (s, 1H, OH), 1.65 (s, 6H, Me).

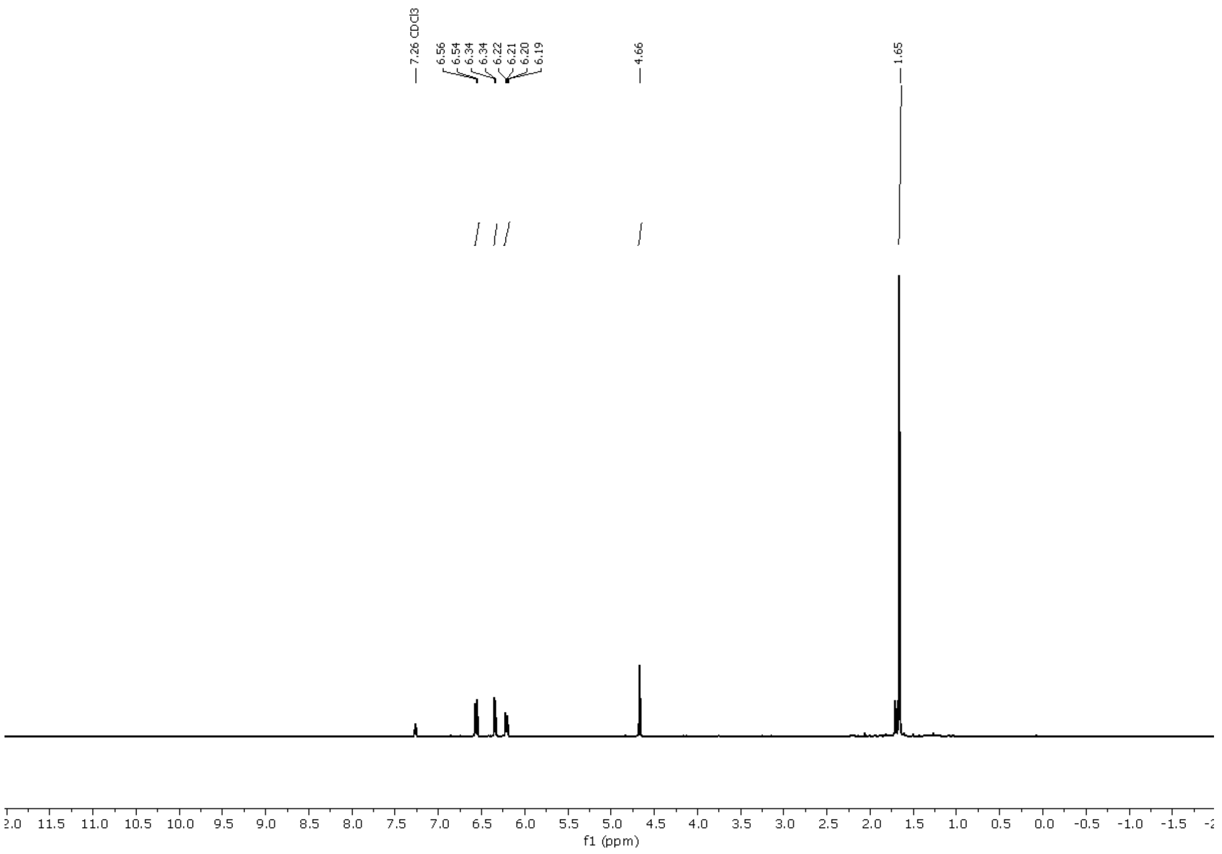

Figure S1.12: ^{1}H NMR (400 MHz, Chloroform-*d*) spectrum of pBTO.

2,2-dimethyl-5-(pentyloxy)benzo[d][1,3]dioxole (p5-BDO) – brown liquid

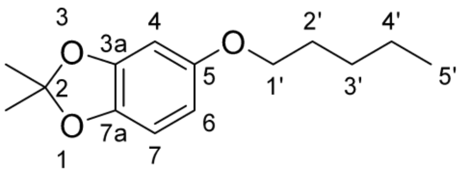

^{1}H NMR (400 MHz, Chloroform-*d*) δ 6.60 (d, *J* = 8.4 Hz, 1H), 6.40 (d, *J* = 2.5 Hz, 1H), 6.27 (dd, *J* = 8.4, 2.5 Hz, 1H), 3.86 (t, *J* = 6.6 Hz, 2H), 1.74 (p, *J* = 6.8 Hz, 2H), 1.65 (s, 6H), 1.47 – 1.31 (m, 4H), 0.92 – 0.84 (m, 3H).

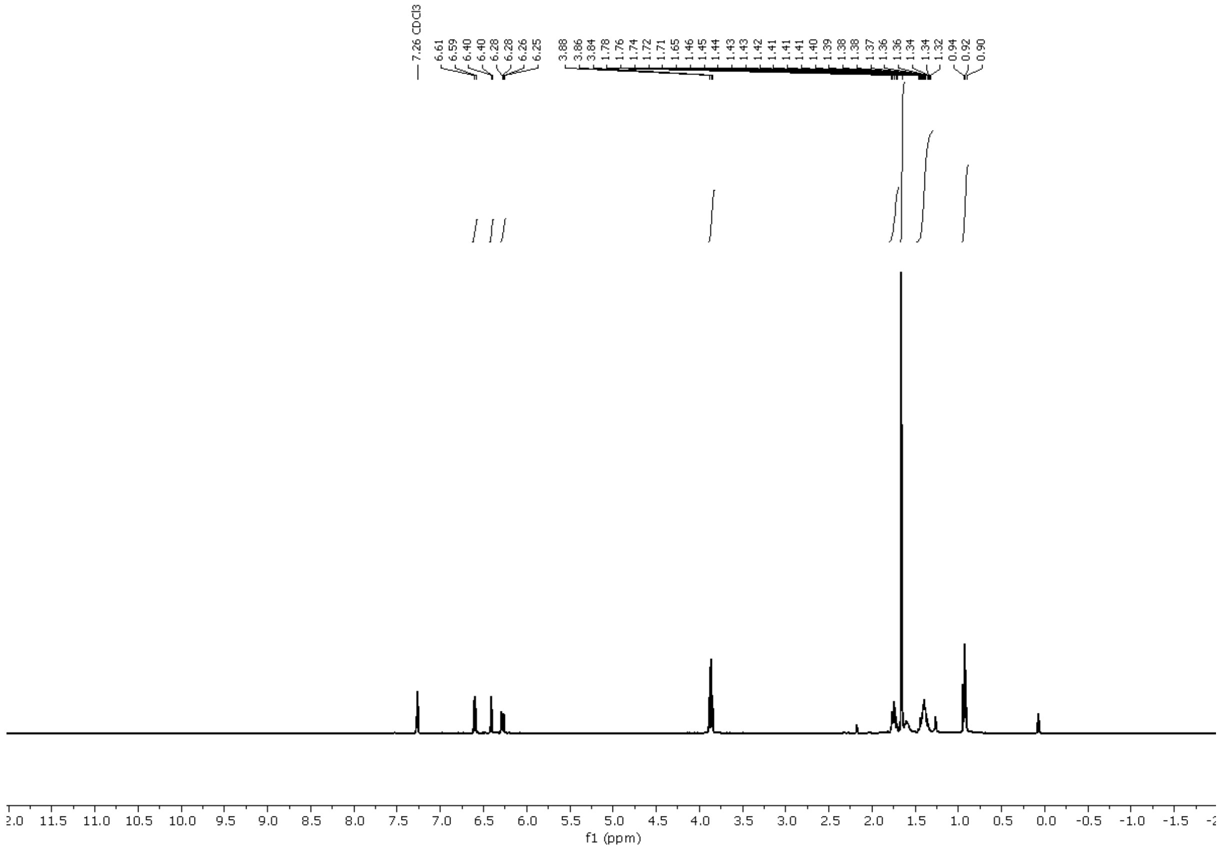

Figure S1.13: ^{1}H NMR (400 MHz, Chloroform-*d*) spectrum of compound p5-BDO.

2,2-dimethyl-5-(hexyloxy)benzo[d][1,3]dioxole (p6-BDO) – brown liquid

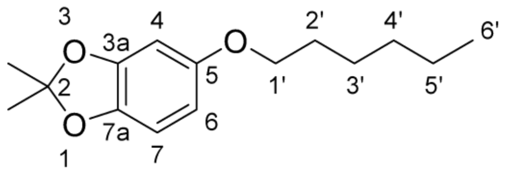

^{1}H NMR (400 MHz, Chloroform-*d*) δ 6.60 (d, *J* = 8.4 Hz, 1H), 6.40 (d, *J* = 2.5 Hz, 1H), 6.27 (dd, *J* = 8.4, 2.5 Hz, 1H), 3.86 (t, *J* = 6.6 Hz, 2H), 1.73 (p, *J* = 6.8 Hz, 2H), 1.65 (s, 6H), 1.43 (p, *J* = 7.2, 6.8 Hz, 2H), 1.38 – 1.28 (m, 4H), 0.96 – 0.84 (m, 3H).

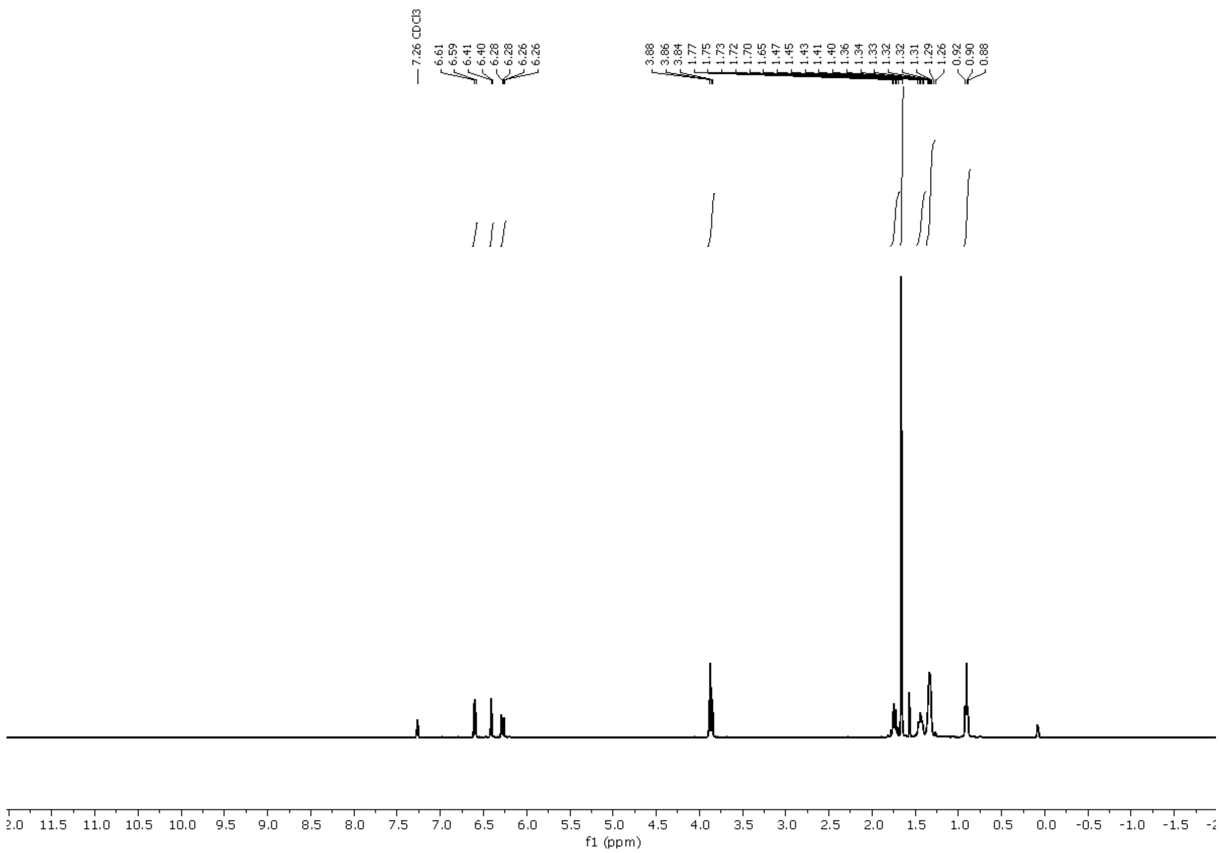

Figure S1.14: ^{1}H NMR (400 MHz, Chloroform-*d*) spectrum of compound p6-BDO.

2,2-dimethyl-5-(heptyloxy)benzo[d][1,3]dioxole (p7-BDO) – brown liquid

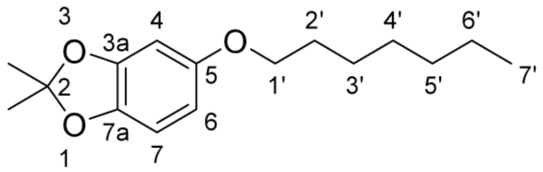

^{1}H NMR (400 MHz, Chloroform-*d*) δ 6.60 (d, *J* = 8.4 Hz, 1H), 6.40 (d, *J* = 2.5 Hz, 1H), 6.27 (dd, *J* = 8.4, 2.5 Hz, 1H), 3.86 (t, *J* = 6.6 Hz, 2H), 1.74 (p, *J* = 6.8 Hz, 2H), 1.65 (s, 6H), 1.42 (q, *J* = 7.3 Hz, 2H), 1.37 – 1.25 (m, 6H), 0.96 – 0.84 (m, 3H).

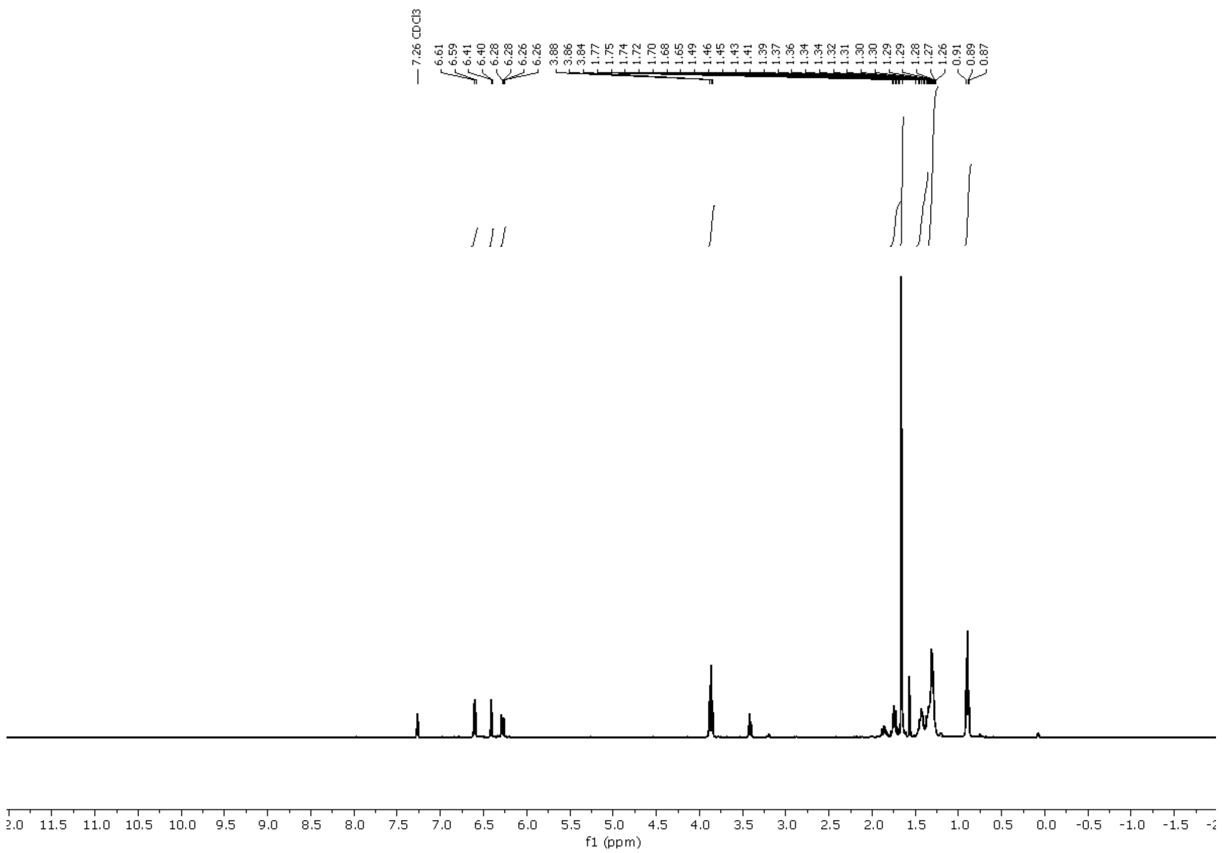

Figure S1.15: ^{1}H NMR (400 MHz, Chloroform-*d*) spectrum of compound p7-BDO.

2,2-dimethyl-5-(octyloxy)benzo[d][1,3]dioxole (p8-BDO) – brown liquid

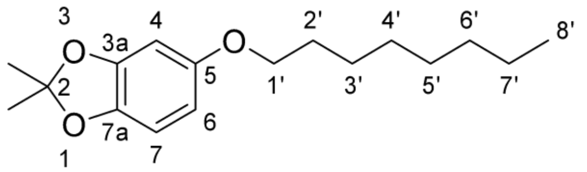

^{1}H NMR (400 MHz, Chloroform-*d*) δ 6.60 (d, *J* = 8.4 Hz, 1H), 6.40 (d, *J* = 2.5 Hz, 1H), 6.27 (dd, *J* = 8.4, 2.5 Hz, 1H), 3.86 (t, *J* = 6.6 Hz, 2H), 1.73 (p, *J* = 6.8 Hz, 2H), 1.65 (s, 6H), 1.43 (dq, *J* = 13.8, 6.6 Hz, 2H), 1.29 (td, *J* = 10.8, 10.2, 6.2 Hz, 8H), 0.96 – 0.84 (m, 3H).

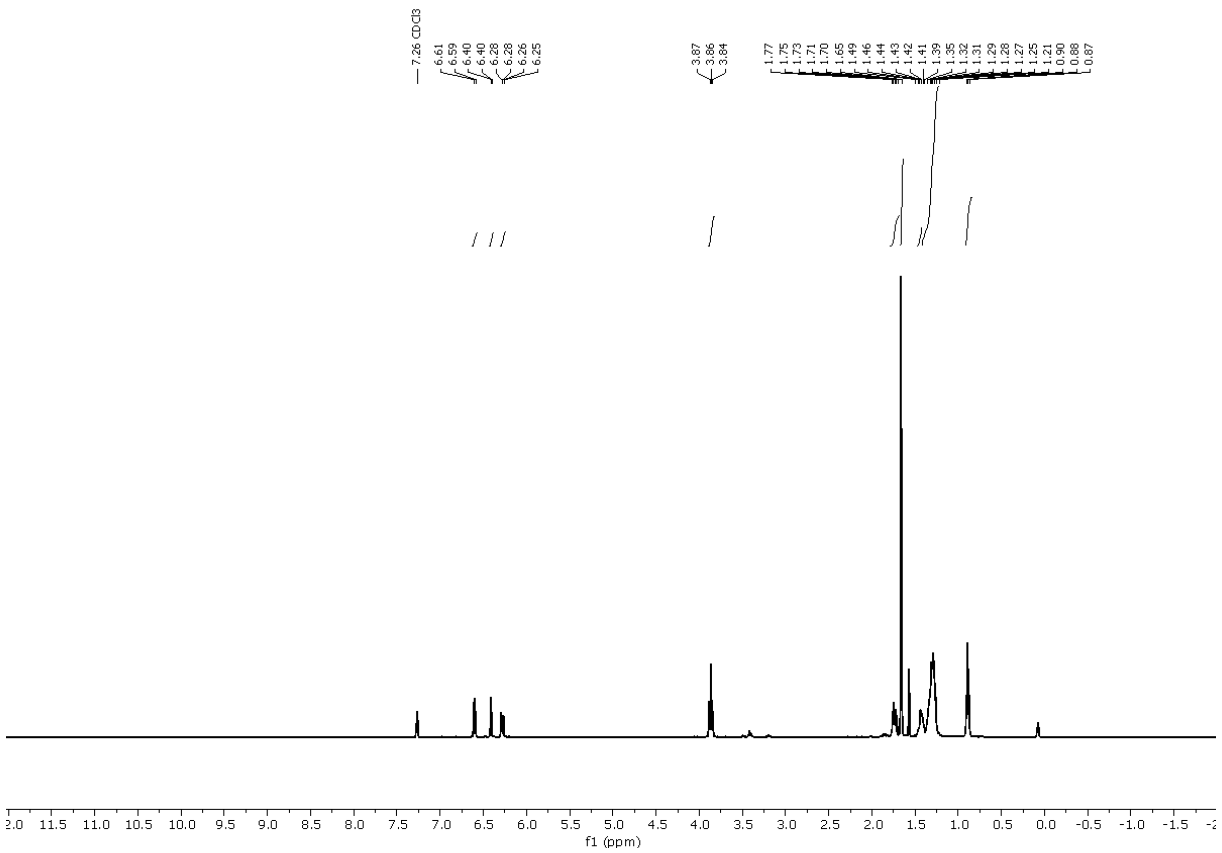

Figure S1.16: ^{1}H NMR (400 MHz, Chloroform-*d*) spectrum of compound p8-BDO.

2,2-dimethyl-5-(nonyloxy)benzo[d][1,3]dioxole (p9-BDO) – brown liquid

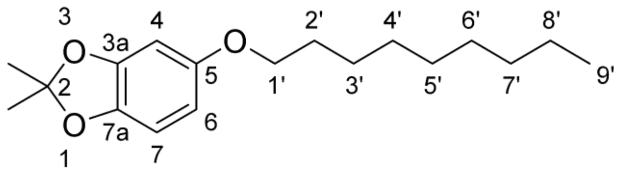

^{1}H NMR (400 MHz, Chloroform-*d*) δ 6.60 (d, *J* = 8.4 Hz, 1H), 6.40 (d, *J* = 2.5 Hz, 1H), 6.27 (dd, *J* = 8.4, 2.5 Hz, 1H), 3.86 (t, *J* = 6.6 Hz, 2H), 1.78 – 1.69 (m, 2H), 1.65 (s, 6H), 1.42 (t, *J* = 7.6 Hz, 4H), 1.38 – 1.21 (m, 8H), 0.92 – 0.84 (m, 3H).

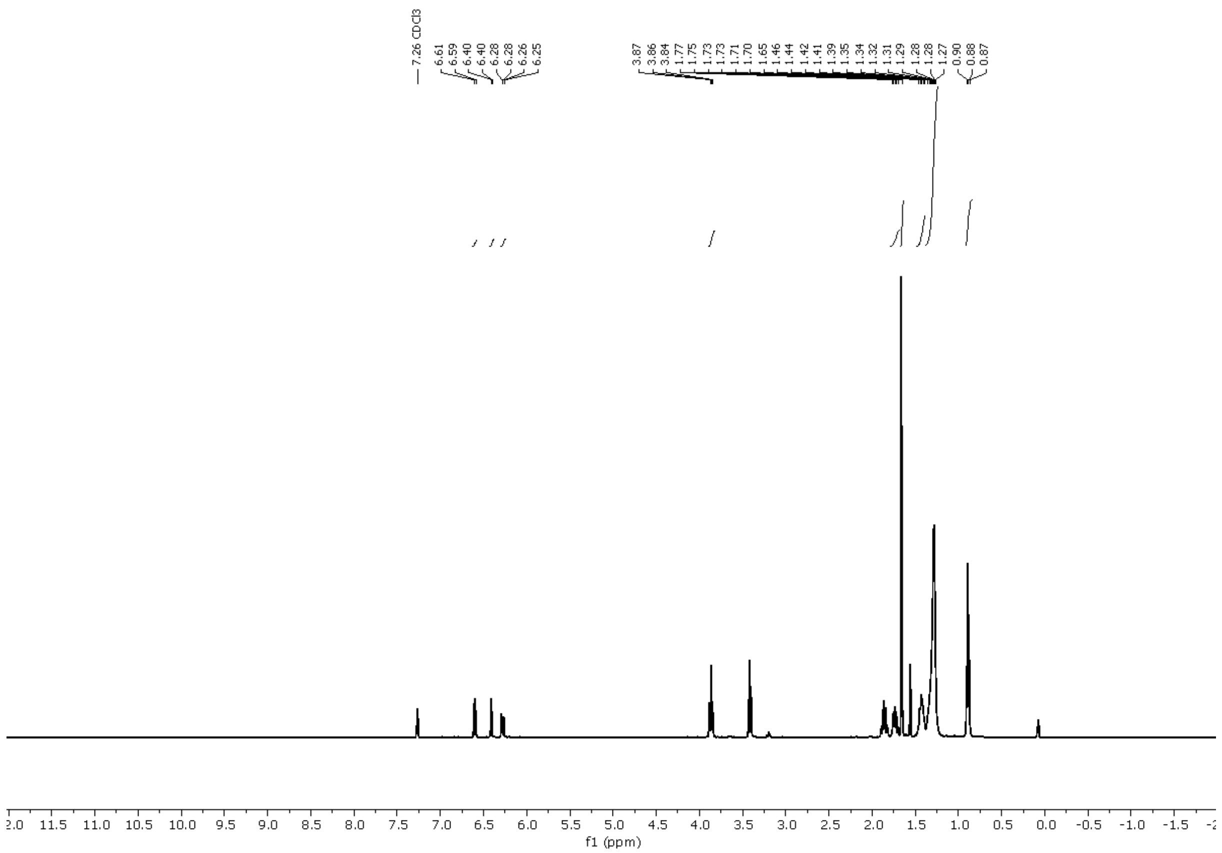

Figure S1.17: ^{1}H NMR (400 MHz, Chloroform-*d*) spectrum of compound p9-BDO.

2,2-dimethyl-5-(dodecyloxy)benzo[d][1,3]dioxole (p12C) – brown liquid

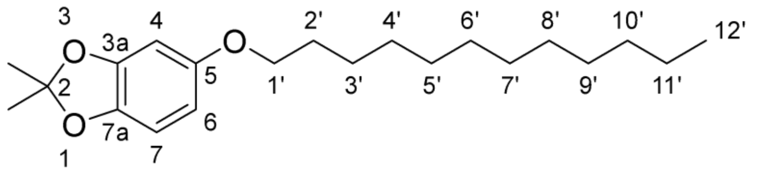

^{1}H NMR (400 MHz, Chloroform-*d*) δ 6.60 (d, *J* = 8.4 Hz, 1H), 6.40 (d, *J* = 2.5 Hz, 1H), 6.27 (dd, *J* = 8.4, 2.5 Hz, 1H), 3.86 (t, *J* = 6.6 Hz, 2H), 1.73 (p, *J* = 6.8 Hz, 2H), 1.65 (s, 6H), 1.42 (t, *J* = 7.6 Hz, 4H), 1.36 – 1.21 (m, 14H), 0.88 (t, *J* = 6.7 Hz, 3H).

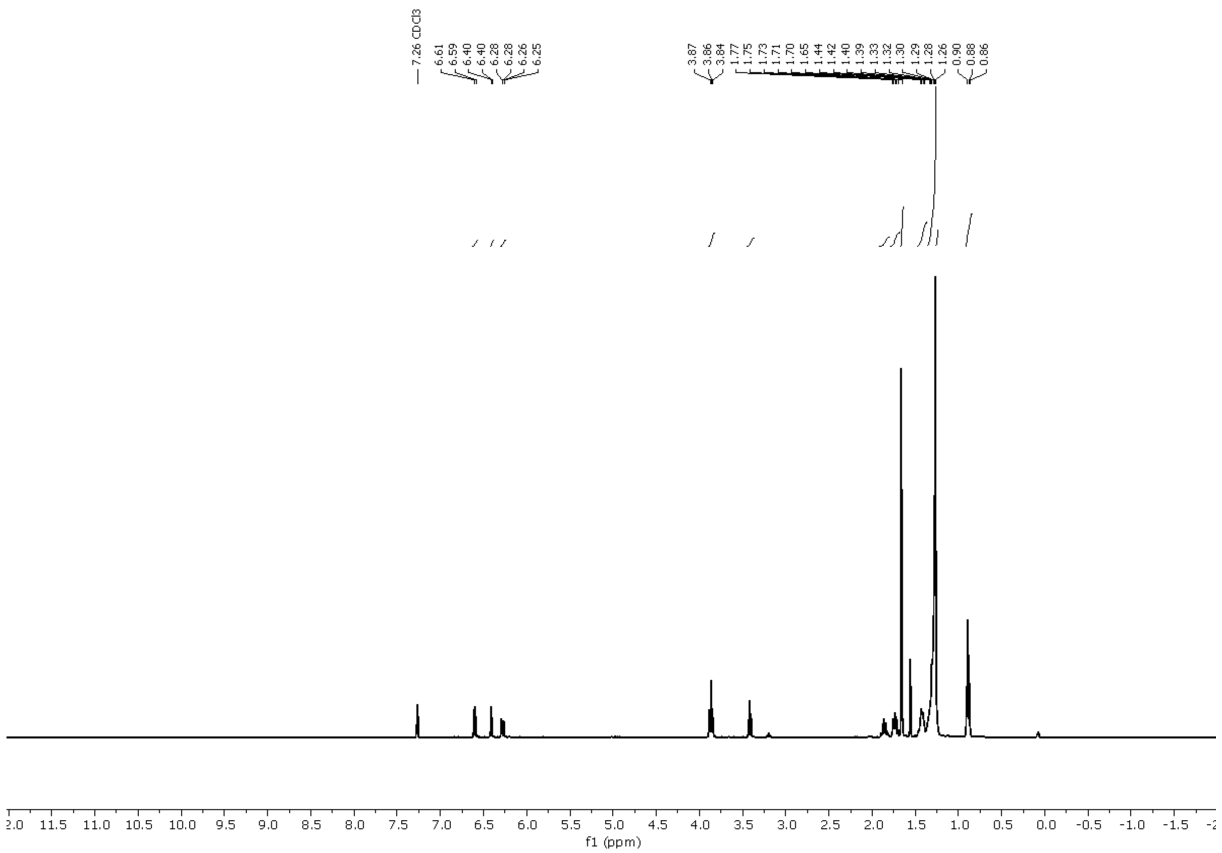

Figure S1.18: ^{1}H NMR (400 MHz, Chloroform-*d*) spectrum of compound p12C.

2,2-dimethyl-5-(tetradecyloxy)benzo[d][1,3]dioxole (p14-BDO) – brown liquid

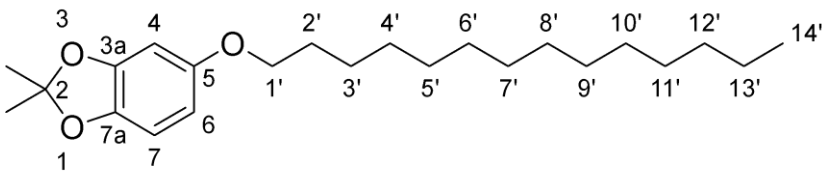

^{1}H NMR (400 MHz, Chloroform-*d*) δ 6.60 (d, *J* = 8.4 Hz, 1H), 6.40 (d, *J* = 2.5 Hz, 1H), 6.27 (dd, *J* = 8.4, 2.5 Hz, 1H), 3.86 (t, *J* = 6.6 Hz, 2H), 1.74 (q, *J* = 7.0 Hz, 2H), 1.65 (s, 6H), 1.42 (t, *J* = 7.6 Hz, 4H), 1.27 (d, *J* = 7.3 Hz, 18H), 0.92 – 0.84 (m, 3H).

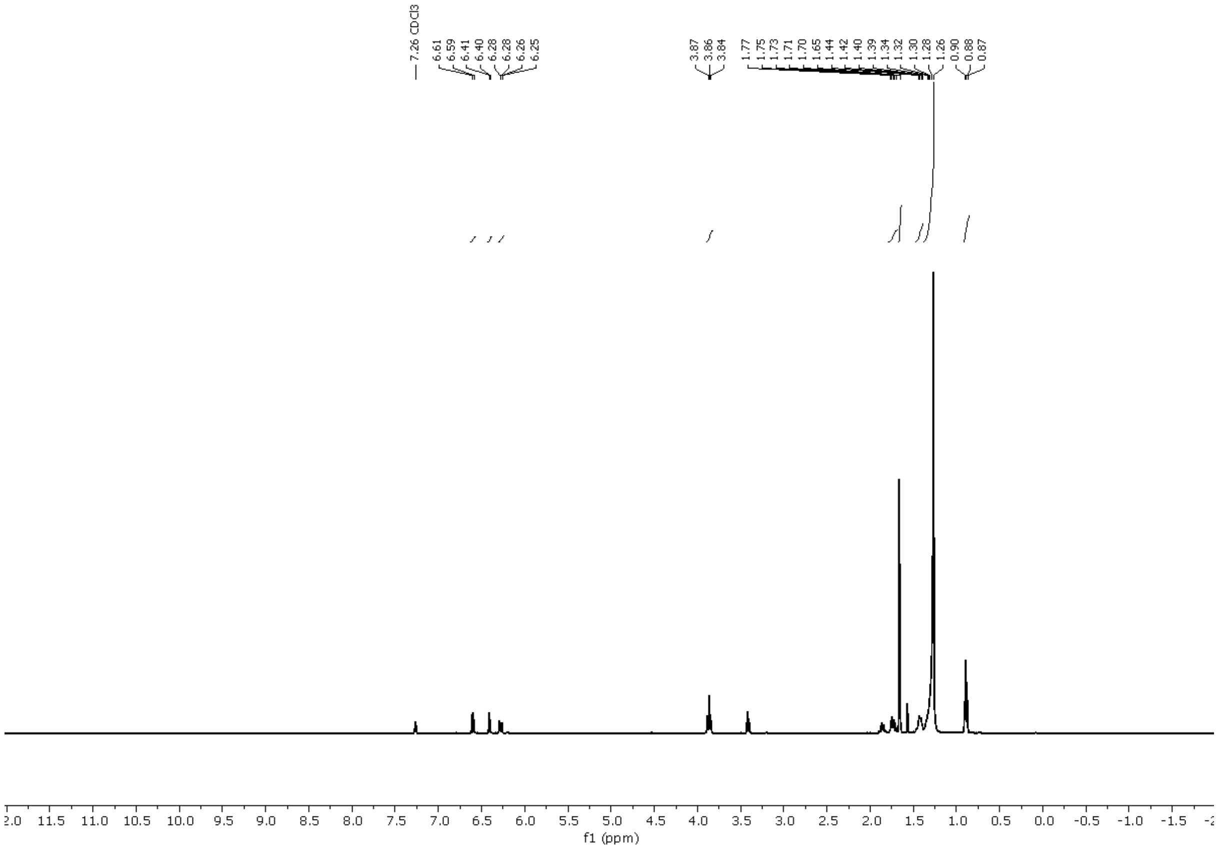

Figure S1.19: ^{1}H NMR (400 MHz, Chloroform-*d*) spectrum of compound p14-BDO.

Section S2 Bacterial growth inhibition

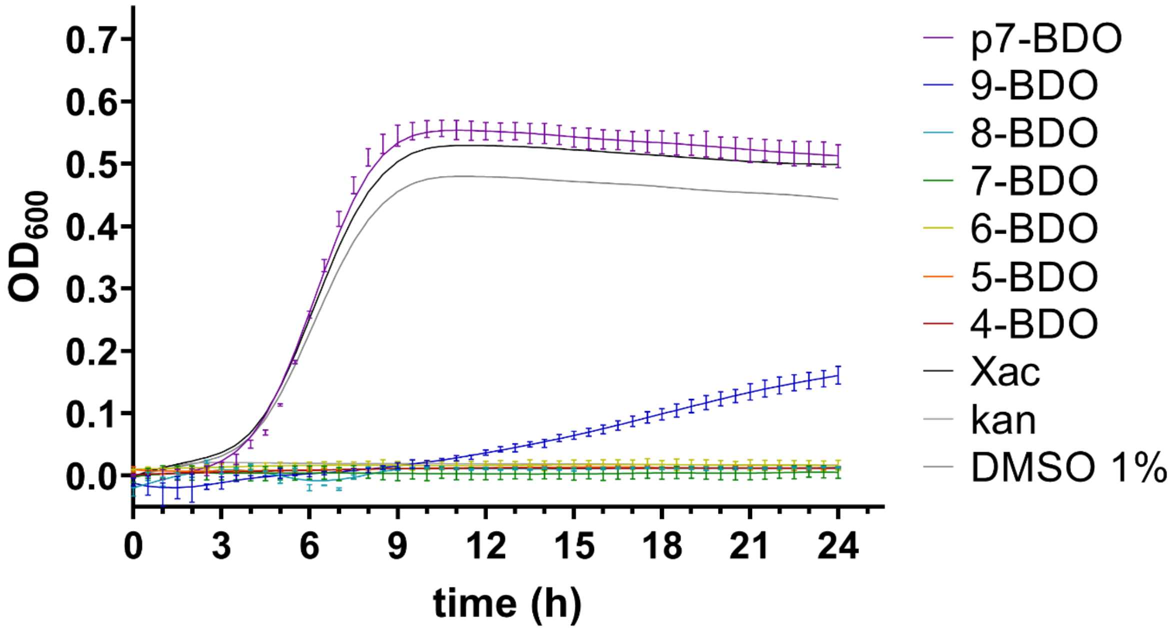

Figure S2.1: *X. citri* growth curves under treatment with alkyl-BDOs at 100 µg·mL^{-1}

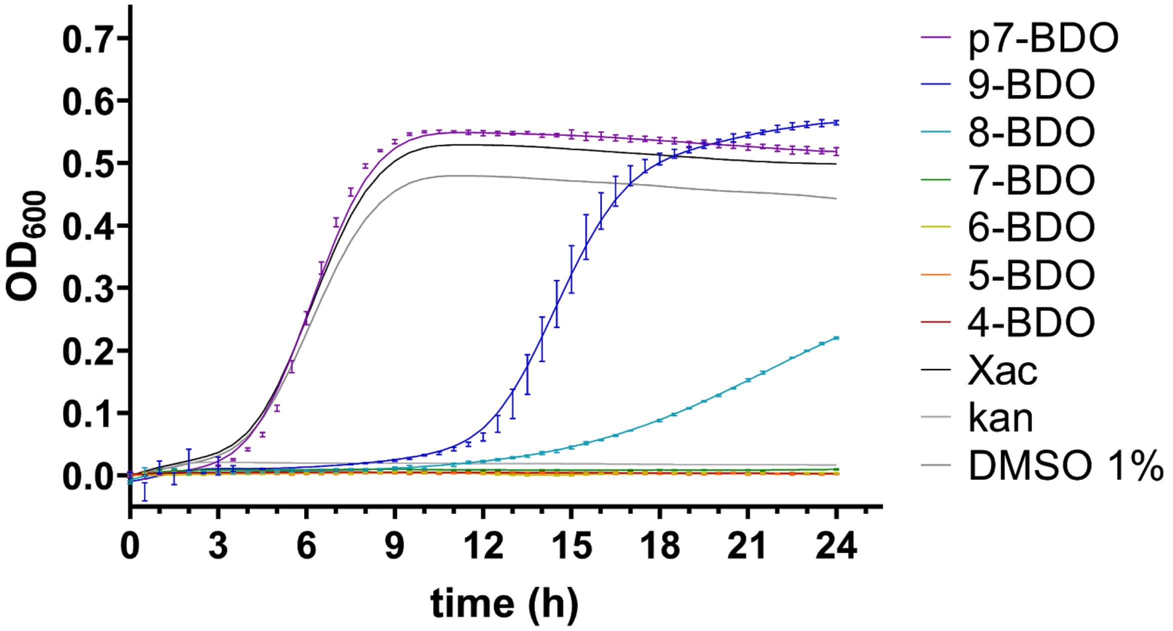

Figure S2.2: *X. citri* growth curves under treatment with alkyl-BDOs at 50 μg·mL^{-1}

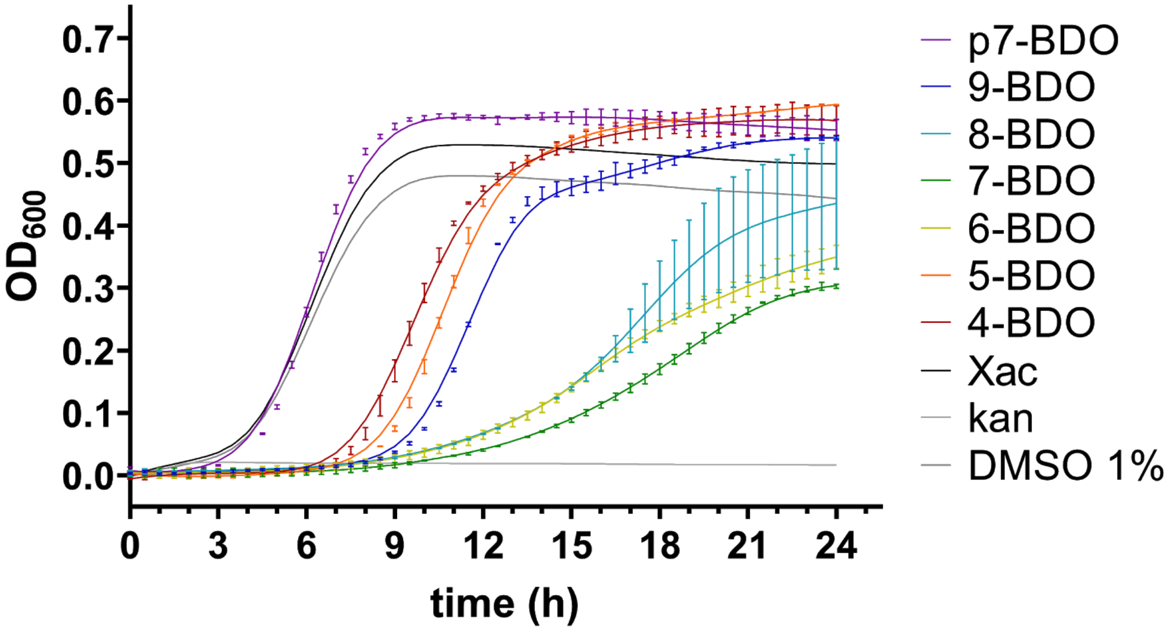

Figure S2.3: *X. citri* growth curves under treatment with alkyl-BDOs at 25 μg·mL^{-1}

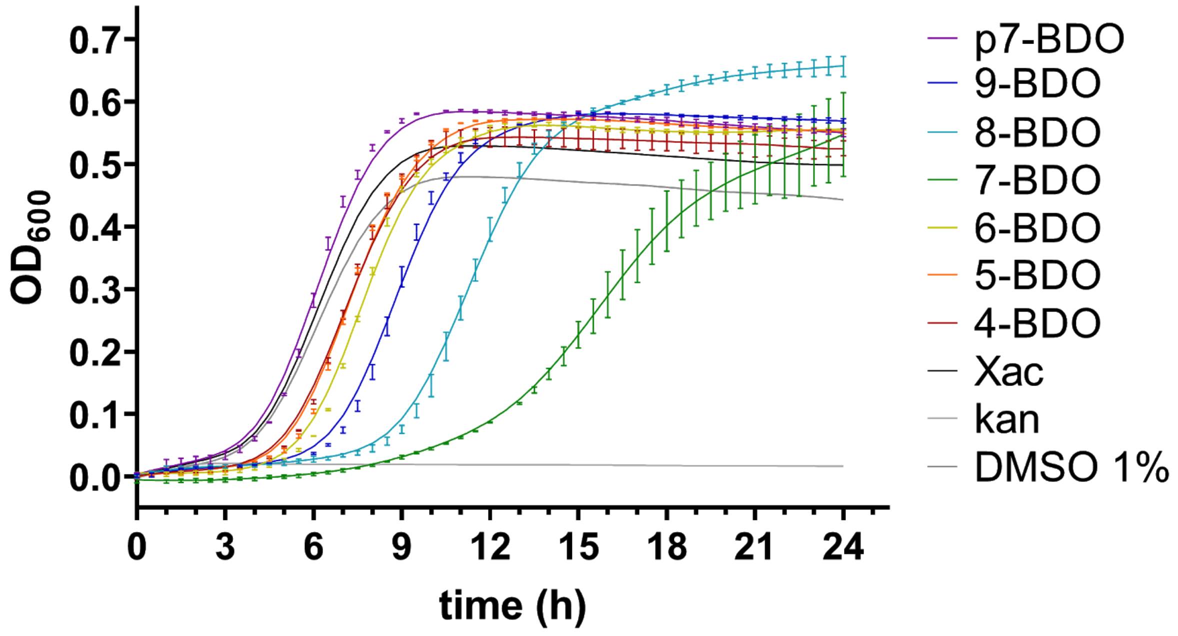

Figure S2.4: *X. citri* growth curves under treatment with alkyl-BDOs at 12.5 μg·mL^{-1}

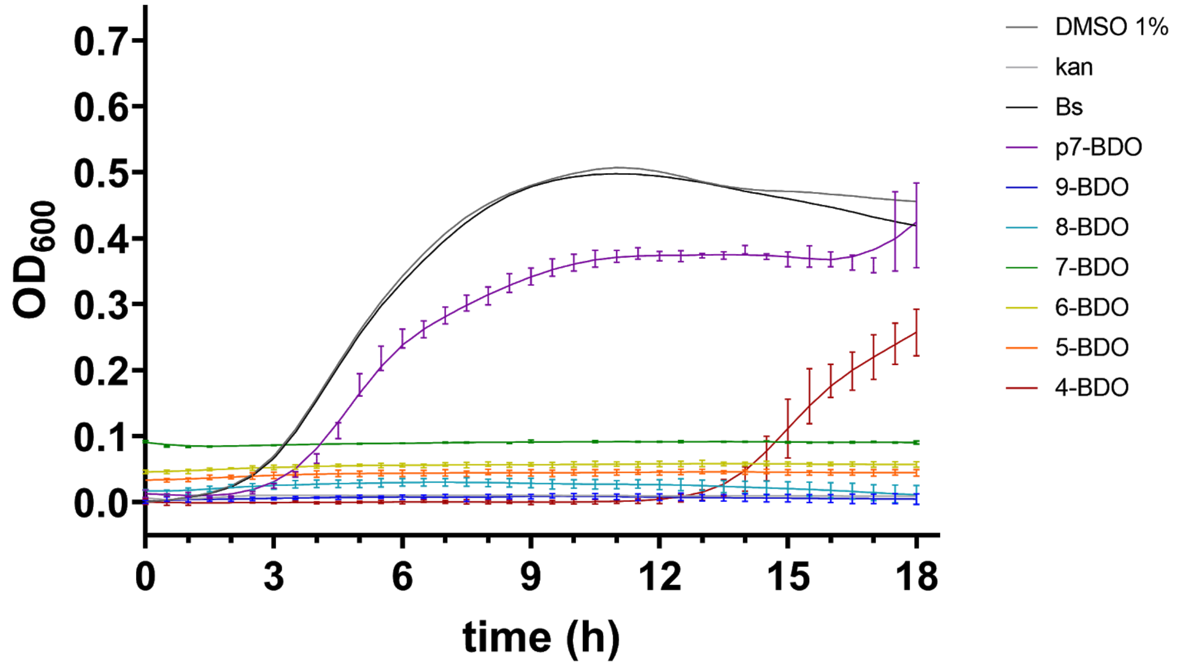

Figure S2.5: *B. subtilis* growth curves under treatment with alkyl-BDOs at 100 μg·mL^{-1}

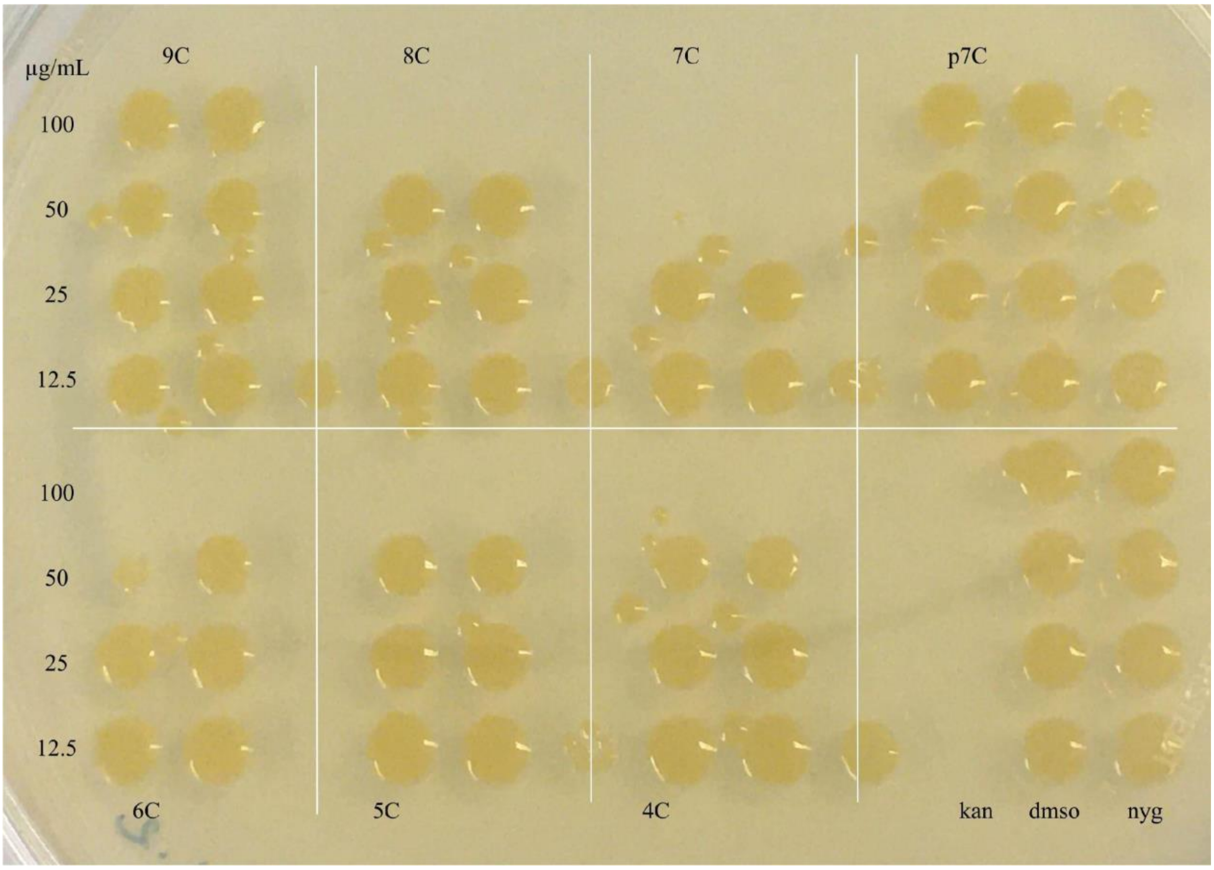

Figure S2.6: *X. citri* MBC plate after treatment with alkyl-BDOs and incubation for 48 h.
